# Molecular mechanisms for dynamic regulation of N1 riboswitch by aminoglycosides

**DOI:** 10.1093/nar/gky833

**Published:** 2018-09-20

**Authors:** Marta Kulik, Takaharu Mori, Yuji Sugita, Joanna Trylska

**Affiliations:** 1 RIKEN, Hirosawa, Wako City, Saitama 351-0198, Japan; 2 Centre of New Technologies, University of Warsaw, Banacha 2c, 02-097 Warsaw, Poland

## Abstract

A synthetic riboswitch N1, inserted into the 5′-untranslated mRNA region of yeast, regulates gene expression upon binding ribostamycin and neomycin. Interestingly, a similar aminoglycoside, paromomycin, differing from neomycin by only one substituent (amino versus hydroxyl), also binds to the N1 riboswitch, but without affecting gene expression, despite NMR evidence that the N1 riboswitch binds all aminoglycosides in a similar way. Here, to explore the details of structural dynamics of the aminoglycoside-N1 riboswitch complexes, we applied all-atom molecular dynamics (MD) and temperature replica-exchange MD simulations in explicit solvent. Indeed, we found that ribostamycin and neomycin affect riboswitch dynamics similarly but paromomycin allows for more flexibility because its complex lacks the contact between the distinctive 6′ hydroxyl group and the G9 phosphate. Instead, a transient hydrogen bond of 6′-OH with A17 is formed, which partially diminishes interactions between the bulge and apical loop of the riboswitch, likely contributing to riboswitch inactivity. In many ways, the paromomycin complex mimics the conformations, interactions, and Na^+^ distribution of the free riboswitch. The MD-derived interaction network helps understand why riboswitch activity depends on aminoglycoside type, whereas for another aminoglycoside-binding site, aminoacyl-tRNA site in 16S rRNA, activity is not discriminatory.

## INTRODUCTION

**Figure 1.**
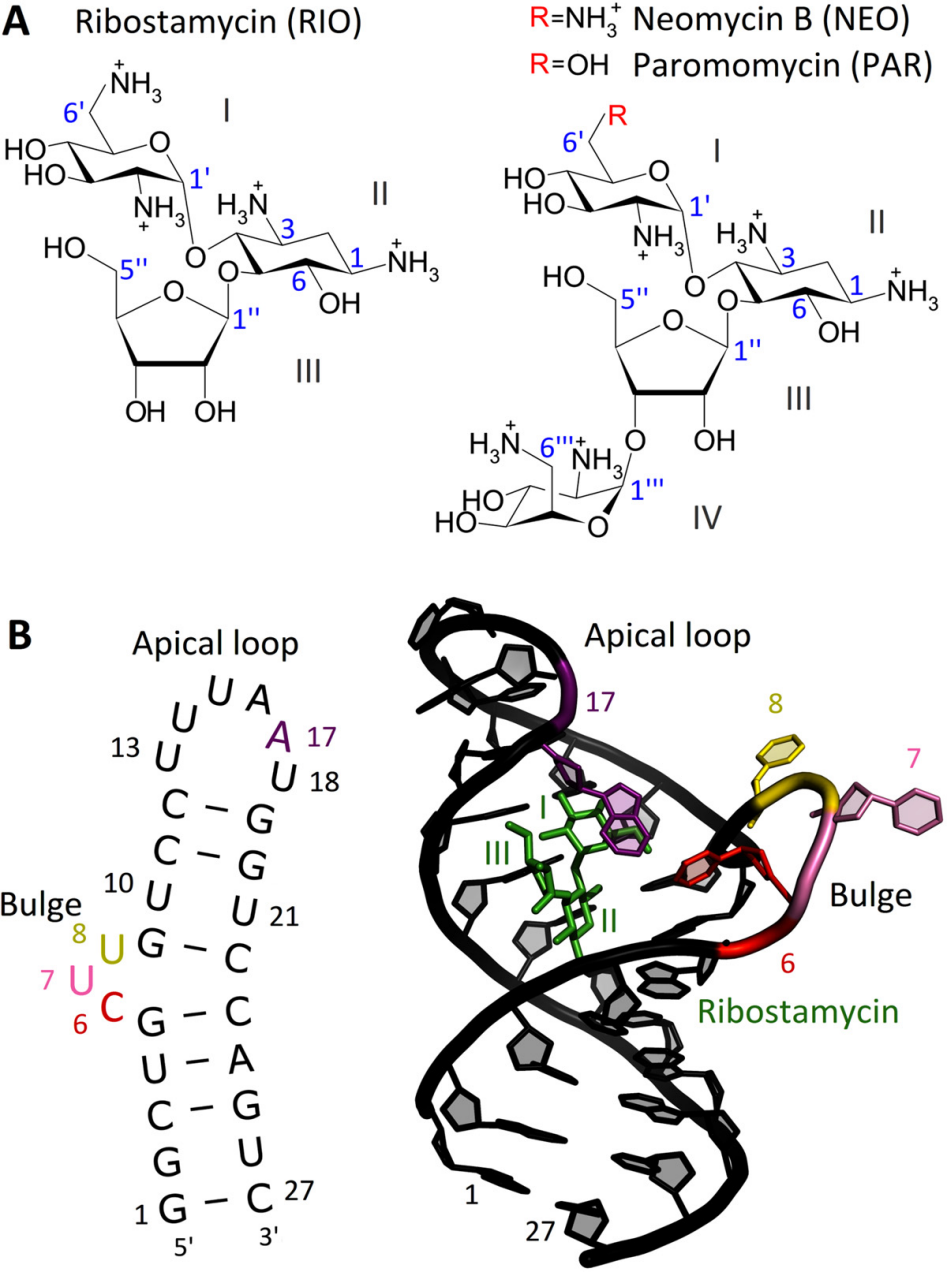
(**A**) Chemical structures of 2-deoxystreptamine aminoglycosides. Numbering of carbon atoms is shown in blue. (**B**) Secondary structure of the N1 riboswitch and visualization of its NMR structure with ribostamycin (PDB ID: 2n0j).

Aminoglycosides are broad-spectrum antibiotics used in the treatment of severe bacterial infections (Figure 1A). Their mechanism of action is to disturb protein synthesis in bacteria by binding to rRNA. The primary binding site of 2-deoxystreptamine (2-DOS) aminoglycosides is the aminoacyl-tRNA site (A-site) located in the small ribosomal subunit. Upon aminoglycoside binding, two A-site adenines (A1492 and A1493) flip out from an internal loop and become locked in a position similar to that observed during mRNA decoding when a cognate aminoacyl-tRNA anti-codon is bound to the mRNA codon. Thus, the aminoglycoside-induced flipped-out state of A1492 and A1493 allows also non-cognate tRNAs to be accommodated and thereby the production of nonfunctional proteins (1).

Aminoglycosides also bind to other RNAs, for example to the aptamer domains of synthetic riboswitches. Although the existence of natural aminoglycoside-sensing riboswitches, regulating the expression of aminoglycoside acetyl transferase and adenyl transferase in bacteria, remains disputable (2,3), various types of natural riboswitches have been discovered in non-coding regions of mRNAs, not only in prokaryotes but also in eukaryotes (4).

The term riboswitch was coined to describe RNAs that ‘control gene expression by binding metabolites without the need for protein factors' (5,6). However, the same term is also used for switches responsive to temperature, cations or small metabolites. Typically, riboswitches are located in the 5′ non-coding region of mRNA and contain a ligand binding sensory domain (aptamer), as well as a downstream expression platform. Specific binding of ligand to aptamer structurally rearranges the platform and controls expression of genes located in the same mRNA. The expression platforms utilize a variety of mechanisms and structural patterns to regulate gene expression, for example via transcription termination or translation initiation in bacteria or splicing control in eukaryotes. As a result, expression platform sequences vary across species, while aptamer sequences are well conserved (4). Riboswitches, especially some of those engineered in eukaryotes, may lack expression platforms, and then regulation is by the aptamer-ligand complex mechanically blocking mRNA scanning and translation initiation (7) (see Supplemental Figure S1).

Riboswitches are unique in that they regulate gene expression in a spatial, temporal and dose-dependent manner (8). However, identification of new riboswitch ligands with useful properties is not straightforward, partly because of insufficient understanding of their mode of action (9). Currently, there is much interest in developing antimicrobials that target riboswitches (10), and a few highly selective modulators have been described, e.g. for the bacterial riboflavin riboswitch (11).

A few years before the discovery of the first natural riboswitch, technology to isolate aptamers had been developed (12). Aptamers are typically selected by screening huge libraries of nucleic acid oligomers for their ability to bind a specific molecule (13). Some of them can replace natural aptamers, and lead to new riboswitches. Such synthetic riboswitches are currently receiving much attention (14) due to their manifold applications, e.g. as biosensors (15).

In 2008, a 27-nucleotide-long N1 riboswitch was found in a screening study for aptamers binding neomycin (NEO) (16). The N1 riboswitch also binds other 2-DOS aminoglycosides similar to NEO (Figure 1B). It is the smallest synthetic riboswitch active *in vivo* (17), and comprises a hairpin with a lower stem (nucleotides 14 and 427) and an upper stem, and two flexible regions - the bulge and apical loop - responsible for riboswitch functionality (Figure 1B).

The N1 riboswitch is active when inserted into the 5′-untranslated region of an mRNA (5′-UTR) of a reporter gene (16) capable of expressing green fluorescent protein. Its mode of action in yeast is based on blocking ribosomal scanning of mRNA upon binding of 2-DOS aminoglycosides (Supplemental Figure S1). Fluorescence measurements showed that green fluorescent protein expression depends on NEO binding to the N1 riboswitch. Mutagenesis has shown that the bulge and apical loop are important for ligand binding and riboswitch activity—the bulge being regulatory and apical loop modulatory (18). Further, the RNA construct that performs best for NEO also works reasonably well for ribostamycin (RIO) (16). Surprisingly, paromomycin (PAR) does not inhibit green fluorescent protein gene expression even though it differs from NEO by only one substituent in ring I (Figure 1 A).

UV-monitored thermal melting and isothermal titration calorimetry studies point to structural changes and thermal stabilization of the N1 riboswitch upon ligand binding (19). Dissociation constants for NEO, RIO and PAR binding to the isolated riboswitch (0.010 ± 0.002, 0.33 ± 0.03 and 5.13 ± 0.26 μM, respectively (20) are consistent with gene regulatory activity *in vivo* (16). NMR structures of the ligand-free riboswitch and in complex with RIO (19) and PAR (20) suggest that an unstructured free state and ligand binding effects on structural dynamics underlie N1 regulatory properties (19–21).

The aim of our study was to investigate the dynamics of the N1 riboswitch in the free and ligand bound states in atomistic detail in order to establish why this riboswitch is biologically active upon binding either RIO or NEO and is inactive upon binding PAR. We applied the technique of molecular dynamics simulations (MD), and, to ensure sufficient sampling of this highly flexible nucleic acid system, we also used temperature replica-exchange MD (REMD) (22). The predictive power of REMD simulations is well described, as for example in the studies of DNA duplex formation (23) and RNA tetranucleotide folding (24). Moreover, the REMD method was previously shown to sample even five times more conformational space than standard MD simulations of comparable total simulation time (25).

The simulations provide detailed descriptions of the dynamics of the N1 riboswitch with the three aminoglycosides. We are able to propose a tertiary structure of the N1 riboswitch in a ligand-free state, as yet experimentally not resolved. We further compare the mode of 2-DOS aminoglycoside binding to the N1 riboswitch with the mode of binding to the ribosomal decoding A-site in the bacterial small subunit. In contrast to the N1 riboswitch, the A-site does not discriminate between PAR and NEO and the biological activities of their complexes are similar.

## MATERIALS AND METHODS

### Structure preparation

The NMR structures of the N1 riboswitch with RIO (termed N1-RIO) and PAR (N1-PAR) were taken from the RCSB Protein Data Bank (PDB IDs 2n0j and 2mxs, respectively (20)). The structures of free riboswitches, i.e. without ligands (termed N1a and N1b), were prepared by removing ligands from the NMR structures. The root mean square deviation (RMSD) between the non-hydrogen atoms of N1a and N1b was 1.7 Å. NEO-riboswitch complex (N1-NEO) was obtained by replacing (in the 2mxs structure) the 6′-OH group of PAR with the ammonium group. The simulations with the U8G mutation were performed by changing U8 to G using the same base plane orientation. For the complete set of simulated systems see Table 1.

At neutral pH, the studied aminoglycosides are fully protonated in solution (26,27) except for the ammonium group attached to carbon atom no 3 of ring II (Figure 1 A). However, it has been shown that at pH ≥ 5.5 this group takes up a proton and becomes positively charged upon aminoglycoside binding to the A-site (28,29). Thus, it was protonated in our simulations.

Aminoglycoside structure optimization and calculations of ESP charges were performed in Gaussian (30). Partial charges were assigned with antechamber of Amber11 (31). All solutes were solvated with TIP3P water, neutralized with Na^+^ ions, and excess Na^+^ and Cl^−^ ions were added to achieve 100 mM ionic strength (all with LEaP from Amber14 (31) and ion parameters from (32)). A shell of at least 20 Å of water molecules was provided around the RNA or aminoglycoside.

The ff14SB and GAFF Amber force field parameters were used for RNA and aminoglycosides. For nucleic acids, the performance of the ff12SB force field, equivalent to ff14SB, was previously validated using the REMD of a tetranucleotide (24). For aminoglycosides, we evaluated the force field by comparing the inter-proton distances from NMR data (33) with the distances measured in MD trajectories of free aminoglycosides (Supplemental Table S1 and Figure S2).

### MD and REMD

Energy minimizations and simulations were carried out with the SPDYN module of the GENESIS (v. 1.0 and 1.1) suite of programs (34,35). Electrostatic interactions were calculated with the particle mesh Ewald method (36) with 72 or 80 gridpoints along every dimension. Non-bonded interactions were truncated at 12 Å using 13.5 Å Verlet pair list distance. Dispersion correction for van der Waals interactions was applied. Water molecules were treated as rigid bodies with the SETTLE algorithm (37). Energy minimization was performed using 5000 steps of the steepest descent algorithm with 10 kcal/(mol·Å^2^) harmonic restraints on heavy atoms of aminoglycosides and RNA. The systems were equilibrated in the NVT ensemble for 2.5 ns with positional restraints gradually decreased every 500 ps. Before the MD production stages, further equilibration without any restraints was conducted for 1 ns in the NPT ensemble at 310.15 K. The final procedure preceding REMD was based on structures after equilibration with restraints, which were again minimized and equilibrated (50 ps with 5 kcal/(mol·Å^2^) restraints and 100 ps without restraints per replica) in the NVT ensemble using the average box size obtained from MD simulations at 310.15 K. Leapfrog integrator with a 2 fs time step using the SHAKE algorithm (38) for the bonds involving hydrogen atoms and Langevin temperature/pressure control (39) were applied. In REMD, 32 copies of the system with temperatures exponentially distributed between 298.15 and 370.01 K were used. The production simulations are summarized in Table 1. The simulations for the U8G mutants were carried out using single precision calculations on GPU. The frames in REMD trajectories were sorted according to temperature using the remd_convert tool from GENESIS. For analysis, MD trajectories at 310.15 K and REMD trajectories at 311.13 K were used (further referred to as 310 and 311 K, respectively). 311 K was chosen as a representative temperature because it is close to experimental conditions at which the activity of the riboswitch was tested. The level of convergence of REMD trajectories at 311 K was verified by calculating the overlap between the covariant matrices of the two halves of the analyzed REMD trajectories (details in Supplemental Table S2). We also checked that the results, and thus conclusions of this study, are similar if temperature of 298.15 K was selected for analysis.

**Table 1.** A list of MD and REMD simulations together with the PDB IDs of the starting structures used in this study (20)

System	PDB ID	Simulation details
N1-RIO	2n0j	
N1-NEO	2mxs*	MD (100 ns, 310.15 K) and
N1-PAR	2mxs	REMD (32 replicas, 100 ns each,
N1a	2n0j	298.15–370.01 K)
N1b	2mxs	
U8G-RIO	2n0j	
U8G-NEO	2mxs*	REMD (32 replicas, 100 ns each, 298.15–370.01 K)
U8G-PAR	2mxs	
RIO	2n0j	
NEO	2mxs*	MD (100 ns, 310.15 K)
PAR	2mxs	

The star sign denotes the structure in which NEO was built from PAR.

### Data analysis

If not stated otherwise, only heavy (non-hydrogen) atoms of solute were taken for analyses and the first 50 ns of each simulation was omitted. RMSD and root mean square fluctuation (RMSF) calculations, principal component analysis (PCA), overlap of covariant matrices and flipping analysis were performed with Gromacs 5.1 (40). PCA employed Cartesian coordinates of RNA heavy atoms or dihedral angles of RNA backbone: α, β, γ, δ, ε, ζ and glycosidic angle χ. Flipping of RNA bases was quantified by calculating the pseudo-dihedral angles of the centers of masses of selected nucleotides, based on the definition introduced by Song *et al.* (41) (details in Supplemental Figure S3). K-means clustering with a 2 Å clustering radius was performed with the MMTSB Tool Set (42). Ion densities on a 0.5 Å grid and hydrogen bonds between ligands and RNA were calculated with cpptraj (43). MINT (44) was used to analyze hydrogen bonds and stacking interactions within RNA. Hydrogen bond criteria were met if the donor-acceptor distance was ≤3.5 Å and the minimal donor-hydrogen-acceptor angle was 150°. Stacking energy was estimated as a sum of van der Waals and electrostatic force field terms of the stacked nucleobases (44). Figures were prepared with VMD (45), Chimera (46), and Pymol (47).

## RESULTS AND DISCUSSION

### Constant-temperature MD versus REMD simulations

To capture dynamic differences between the complexes with active and inactive N1 riboswitch ligands, we performed REMD simulations, as summarized in Table 1. Although the overall time of the MD and REMD simulations differs significantly, based on our results, we did not expect sufficient sampling gain by extending classical MD. MD simulations tend to get trapped in local energy minima as shown by RMSD fluctuations of RNA. For example, in the N1-PAR complex, the U7 and U8 bases after 25 ns of MD underwent conformational changes that persisted to the end of the simulation (Supplemental Figure S4). REMD, on the other hand, samples conformations sufficiently (including the flipping of the bulge bases) over a broad range of temperatures, preventing such local stabilizations. PCA revealed an enormous difference in the sampling of the dihedral angles of the RNA backbone between MD and REMD (Supplemental Figure S5), the latter providing much wider conformational diversity. PCA performed in Cartesian coordinates (Supplemental Figure S6) showed high flexibility of the free riboswitch. In REMD, for the riboswitch-aminoglycoside complexes, a few separate structural clusters were uncovered. Assuming that each structural cluster corresponds to a basin in the energy landscape, it means that a few new low-energy basins were visited that were not sampled in constant-temperature MD. Therefore, below, we present only the results of REMD simulations.

### Flexibility of N1 riboswitch bulge and apical loop

**Figure 2.**
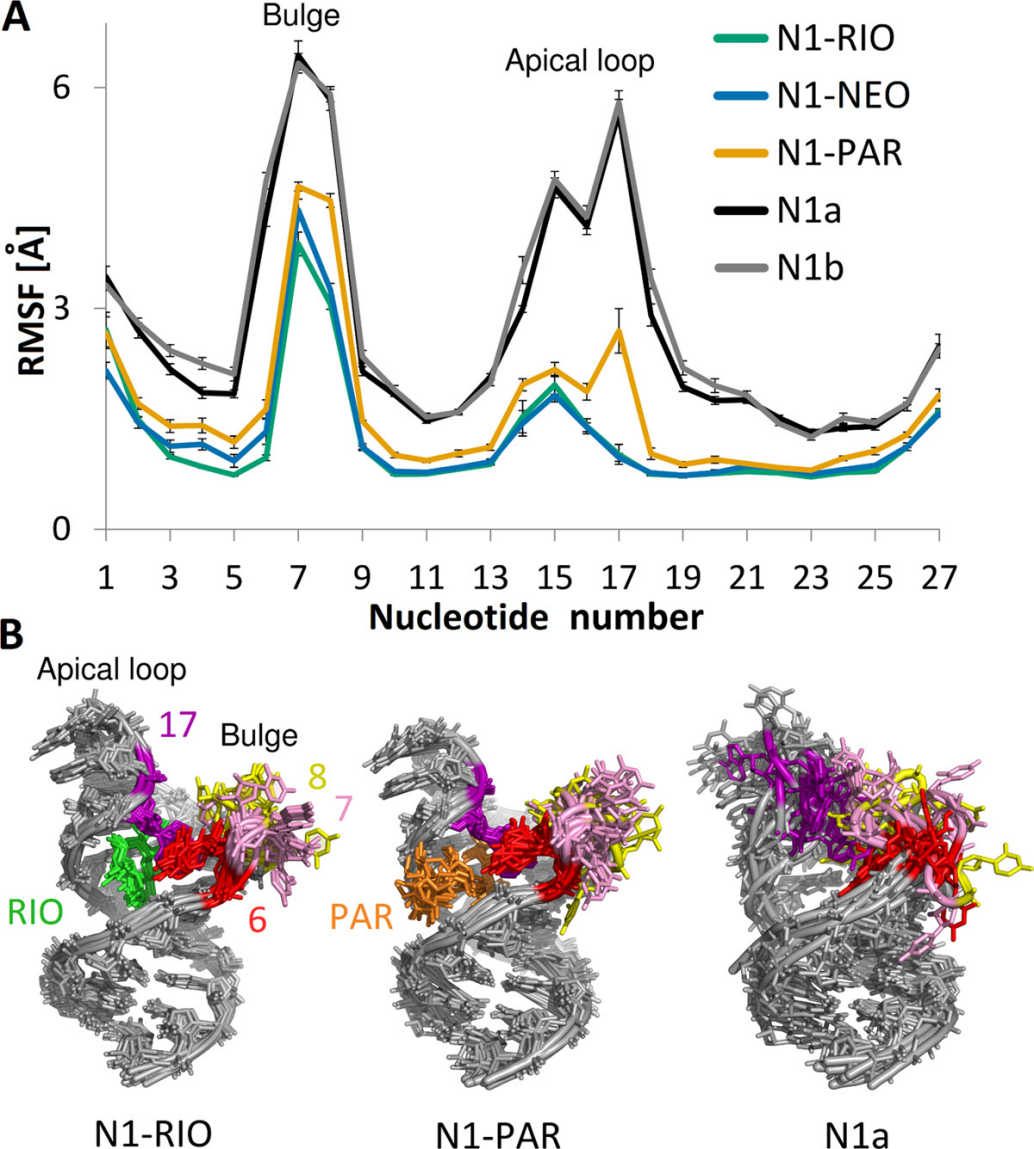
(**A**) RMSF of RNA nucleotides in free riboswitches and their complexes with aminoglycosides. Errors were estimated by block averaging using 10 ns blocks. (**B**) The superposition of the representatives of the first 10 most populated clusters in each system. RMSD-based superposition was performed based on RNA heavy atoms.

The RMSF analysis points to two riboswitch regions that influence its conformational heterogeneity, namely, the bulge and apical loop (Figure 2A). The largest difference in RMSF between the free and complexed riboswitch appears in the apical loop implying that the ligands stabilize this loop. However, for N1-PAR, the RMSF of the apical loop mimics the shape of the free riboswitch. Interestingly, A17 moves freely in the apo-riboswitch, is stable in the complexes with RIO and NEO, and remains mobile in the N1-PAR complex.

K-means clustering analysis shows that the free riboswitch displays the highest structural plurality and also a fairly flat distribution of the number of structures in each cluster (Supplemental Figure S7). The first six most populated clusters of N1a or N1b reflect only 10% of all REMD conformations of the free riboswitch, whereas for the complexes, they reflect more than 50% of conformations. For the complexes, the most populated clusters are similar to one another, but they differ from the free riboswitch (Figure 2 B). Thus, the riboswitch is significantly stabilized by the ligands.

Despite the bulge and apical loop, which are two flexible regions without Watson–Crick (WC) base pairing, the free riboswitch structure is not easily disrupted even at 370 K (Supplemental Figure S8). The RNA backbone preserves almost the same WC hydrogen bond set in the free and complexed N1 states as shown in Supplemental Figure S9 and Table S3. The main differences are that in the complexes U13 and U18 of the apical loop form a *cis* WC/WC base pair, which in the free riboswitch is mostly disrupted and another uracil pair between U10 and U21 is formed via the WC/WC edge. However, the latter pair also shifts, which will be described further.

**Figure 3.**
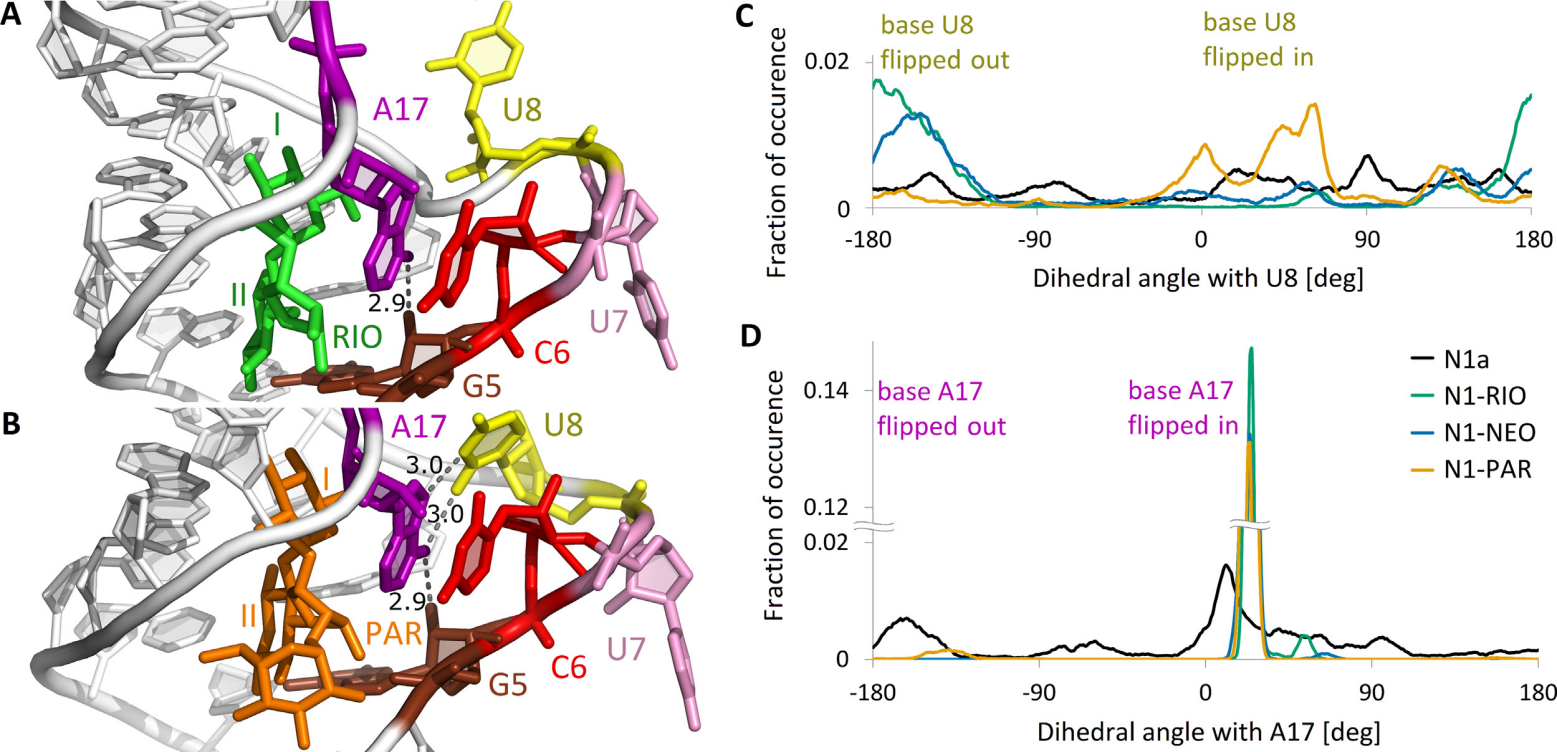
Bases in the bulge region. (A, B) Non-WC base pairs in (**A**) RIO and (**B**) PAR complex. The structures represent two highly populated clusters. Selected distances between heavy atoms in Å units. (C, D) Flipping of (**C**) U8 and (**D**) A17 bases in the simulations shown as distributions of the pseudo-dihedral angles containing these bases (for definition, see Supplemental Figure S3). The graphs are smoothed with running average over 5 consecutive points.

The riboswitch has a stacked lower stem and a short upper stem between the bulge and apical loop, visible as red and orange regions in Supplemental Figure S10. The region deprived of stacking includes the bulge bases, especially U7 located in an extra-helical conformation in the NMR structures. In the simulations of the free riboswitch, we found several transient stacking interactions, confirming its high flexibility (Supplemental Table S4). In all complexes, bases C6 and A17 are stacked in at least 90% of frames (Figure 3A and B). This interaction is barely observed in the free riboswitch suggesting that it is induced by aminoglycoside binding.

The bound and unbound forms of the riboswitch differ most in the non-WC hydrogen bond network, especially for G5 and A17. Figure 3A and B depicts the most frequent interactions in the bulge of N1-RIO and N1-PAR (for complete list, see Supplemental Table S5). In all complexes, we observed a stable hydrogen bond, involving the sugar edge of G5 and either WC or Hoogsteen edge of A17. Only the N1-PAR complex showed relatively frequent non-WC hydrogen bonds between A17 and U8. Different hydrogen bonding and stacking interactions of U8 induce alternative patterns of base flippings in the bulge. The RMSF points to increased fluctuations of U8 in N1-PAR (Figure 2 A) and indeed in this site, U8 acquires different conformations. To quantify the movement of the bulge bases, we analyzed a pseudo-dihedral angle, consisting of four centers of masses (for definition, see Supplemental Figure S3). The angles within the approximate range (−50°, +50°) indicate a flipped-in position of the base, whereas angles around ±180° denote flipped-out. Analyses of pseudo-dihedral angles within the bulge region show that in N1-PAR the U8 base is frequently flipped-in and often positioned towards A17 base, as depicted in Figure 3B and C. This conformation is not present in the NMR structure, where both U7 and U8 bases are always flipped out. In the complexes, base A17 is typically flipped in, as suggested by the narrow peaked distribution of the pseudo-dihedral angles in Figure 3D, and stacked with base C6. Interestingly, in the PAR complex, rare events of a flipped-out conformation of A17 were observed that did not occur in the other complexes and were not revealed in any of the NMR models.

**Figure 4.**
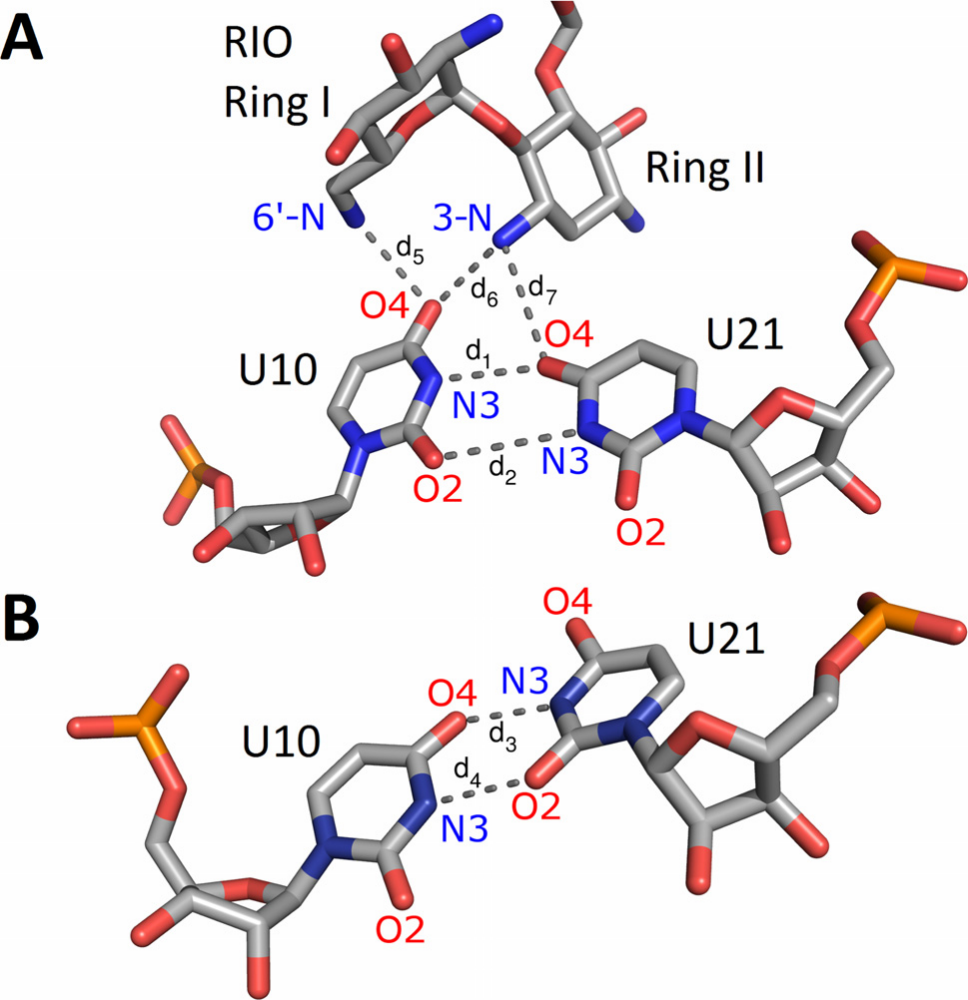
Typical conformations of U10 and U21 with and without aminoglycosides. (**A**) Conformation I in N1-RIO. (**B**) Conformation II in free riboswitch (N1b). Changes in distances *d*_1_–*d*_7_ as a function of the simulation time are provided in Supplemental Figure S11.

The bulge and apical loop are not the only riboswitch regions affected by the aminoglycosides. In all complexes, we observed that the U10–U21 pair stabilizes in one position due to aminoglycoside binding (Figure 4A). A stable hydrogen bond network appears between the aminoglycoside 3-N ammonium group and O4 oxygens of both uracils. Additionally, U10:O4 hydrogen bonds with the 6′-N ammonium group of NEO and RIO. For PAR, the hydrogen bond with the 6′-O hydroxyl is transient (Supplemental Figure S11). In the absence of aminoglycosides, the WC-edge hydrogen bonds between U10 and U21 often shift from conformation I to conformation II as shown in Figure 4B. Similar observations on the conformations of the U10–U21 pair were also made in recent simulations of the N1 riboswitch with and without RIO (48).

### Interactions around 6′ group of aminoglycosides

Simulations indicate that aminoglycosides are anchored inside the riboswitch through several stable direct and indirect (water-mediated) hydrogen bonds. Direct hydrogen bonds, that are identical in all complexes, include contacts with G19 (Supplemental Figure S12). The hydrogen bond between the G19 base and 3′-OH of ring I appears in more than 60% of frames. Also, the hydrogen bonds between ring III and G19 phosphate oxygen are present on average 70% of the time. Ring II interacts with bases G20 and U21, and also G5 in the RIO complex. The 2”-OH group of all aminoglycosides forms stable direct contacts with the phosphate group of G5. We also observed frequent water bridges between aminoglycoside ring II and U4.

**Figure 5.**
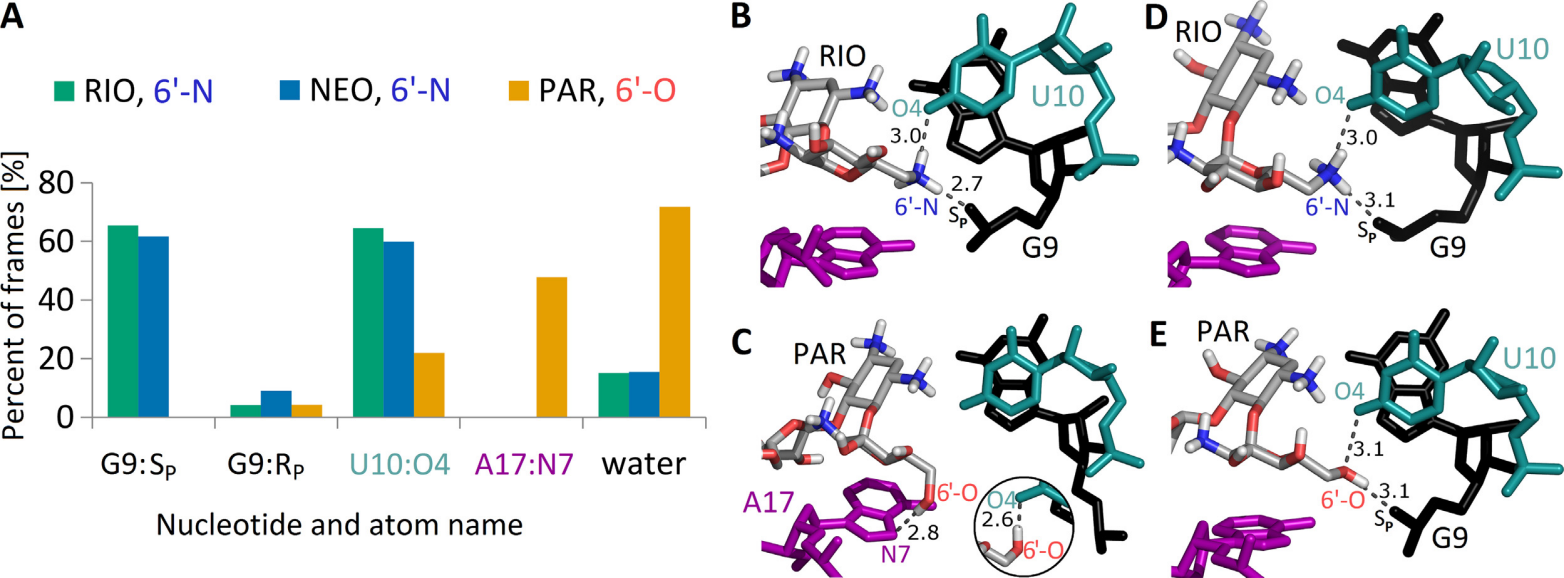
Hydrogen bonds created by aminoglycoside 6′ group. (**A**) The percentage of frames in which the most frequent contacts with either riboswitch atoms or water molecules are present during last 50 ns of trajectories. Only contacts occurring in more than 5% of frames in any simulation are shown. (**B**, **C**) Frequent hydrogen bonds between RIO or PAR and RNA in REMD simulations. The distances shown in Å are measured between non-hydrogen atoms in the cluster representative structures. The inset shows the second most common contact of PAR 6′-OH group, namely with U10:O4 (see A). (**D**, **E**) The same site in NMR structures (PDB IDs 2mxs and 2n0j).

Rings I–III in NEO and RIO are chemically identical. PAR in ring I has a hydroxyl group in the position of the amino group, connected to the 6′ carbon atom (Figure 1). This change from a hydroxyl (in PAR) to amino group (in NEO) largely affects the hydrogen bond network, presented in Figure 5. In RIO and NEO complexes, a hydrogen bond between the 6′-N of aminoglycoside and U10:O4 is present for about 60% of the simulation time. Other protons hold additional polar contacts with the G9 phosphate group, which are not observed in the complex with PAR. Instead, in the PAR complex, 6′-OH interacts with A17:N7. However, this hydrogen bond is not stable and was not detected in the NMR derived-structure (20). The instability of this bond fits with the higher mobility of PAR 6′-O in comparison with 6′-N of other aminoglycosides (Supplemental Figure S13).

**Figure 6.**
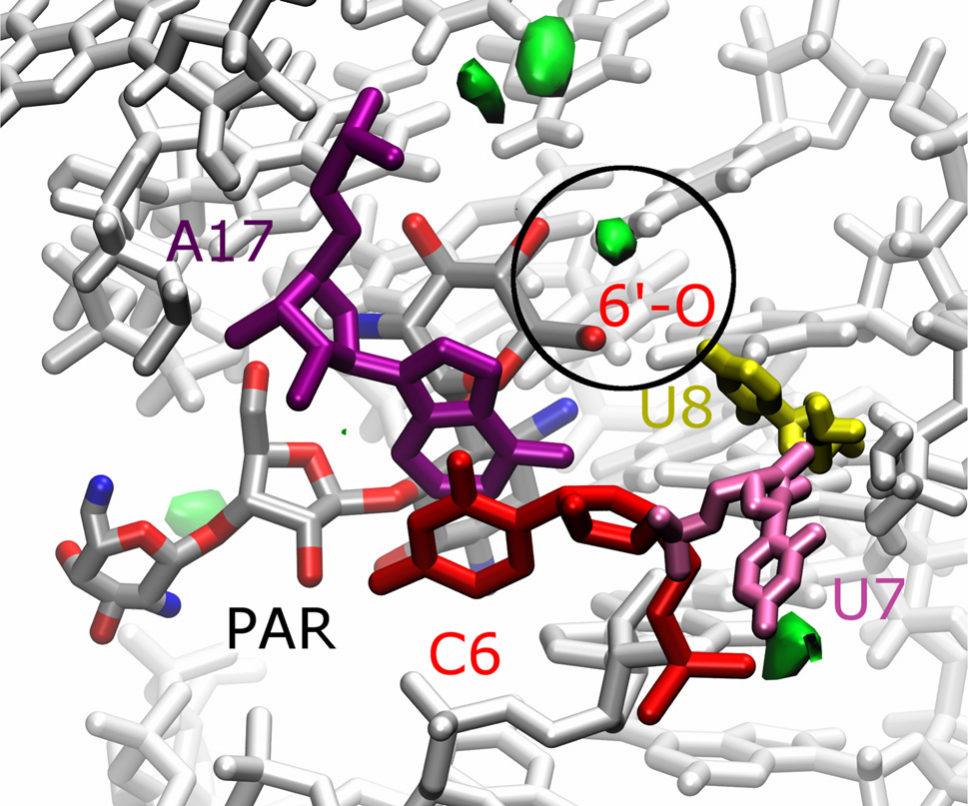
Ion densities in N1-PAR complex. Areas of high Na^+^ concentration (≥0.02 ions/Å^3^) are shown in green. The structures of RNA and aminoglycoside are averaged over the trajectories. A spot of high Na^+^ density in the region of 6′-OH group of PAR is circled.

The REMD simulations were performed using 100 mM NaCl to best imitate experimental conditions. The simulation-derived residence time and spatial arrangement of ions show that Cl^−^ ions, as expected, stay outside the riboswitch due to the electrostatic repulsion of the RNA phosphates. Na^+^ ions, however, penetrate the RNA complexes. Larger patches of high density of Na^+^, shown in Figure 6 and Supplemental Figure S14, occupy similar positions in all complexes, except in the vicinity of ring III in RIO and ring I in PAR. In RIO, the Na^+^ density mimics the location of ring IV present in other aminoglycosides. In PAR, a small Na^+^ dense area appears near the 6′-OH group. Its presence seems to balance the positive charge at this site that is present in the free riboswitch (Supplemental Figure S14). In the other complexes, this region is occupied by the positively charged group 6′-NH$_3^+$ of RIO and NEO. Together with increased Na^+^ density near the 6′-OH group, this region in the N1-PAR complex is also more hydrated (Figure 5A).

### Structure–dynamics–function relationship

In REMD simulations the riboswitch complexes with RIO and NEO are the most stable (Figure 2). The hydrogen bond network created by the 6′-N ammonium group is well maintained (Figure 5B). However, in the same site in the PAR complex, the contacts are strikingly different. The 6′-O hydroxyl forms only transient hydrogen bonds and with various acceptors (Figure 5C). The behaviour of 6′-OH in trajectories of replicas during their random walk in the temperature space is shown in Supplemental Figure S15A and B. The most frequent, observed in 24 out of 32 replicas, termed Scenario 1 (shown in Movie S1), is as follows. In the beginning, the 6′-OH only transiently hydrogen bonds with the G9 phosphate and soon switches to hydrogen bond with either U10:O4 or A17:N7. The 6′-OH group switches between these hydrogen bonds until the end of the simulation. In the meantime, the phosphate group of G9 drifts away to a distance of ∼10 Å from 6′-OH, which triggers base U8 to flip inside the bulge. The N6 amino group of A17 serves as the hydrogen donor to G5 and, occasionally, to U8 (Supplemental Figure S15C). In another sequence, Scenario 2 (Movie S2), that was observed in only 2 out of 32 replicas, the bond between G5 and A17, shown in Figure 3B and Supplemental Figure S15D, breaks and the A17 base wanders off toward other apical loop bases (after rearrangement of stacking interactions involving bases C6 and U8). This kind of riboswitch opening does not occur in either RIO or NEO complexes, but is characteristic of the free riboswitch. Thus, we propose that these A17 flipping-out events are connected with riboswitch activity. These events are not captured in MD simulations at constant temperature.

Further, to confirm the observations of the formation of the U8-A17 hydrogen bond in the PAR complex (Figure 3), not observed in other complexes, we performed the REMD simulations of the riboswitch complexes with U8 replaced by G (Table 1). This U to G replacement changes the base edges and in the PAR complex should lead to breaking the contacts of the base no 8 with A17, but should not affect the dynamics of other complexes. Indeed, in our simulations the hydrogen bond between the WC edge of G8 and Hoogsteen edge of A17 is absent (Supplemental Table S6) even though G8 can still flip into the bulge as indicated by the pseudo-dihedral angle distribution (Supplemental Figure S16). The reason of the flipped-in position of G8 is the same as for the N1-PAR complex (described in Movie S1), namely the 6′-OH and G9 phosphate oxygen contact breaks, the shape of the bulge changes, and G8 flips in. However, once in a flipped-in conformation, G8 prefers to interact with the phosphate oxygens of U10 and U11 and not the A17 base.

Interestingly, experiments in the yeast system have shown that the activity of the riboswitch-NEO complex, measured based on the green fluorescent protein expression levels, is similar with and without the U8G mutation (18). Indeed, in the U8G-NEO, and also in the U8G-RIO complexes, G8 is frequently positioned outside the bulge, in a similar way as U8. Thus, in these complexes the U8G mutation is not expected to change the riboswitch activity (Supplemental Figure S16). The 6′-N aminoglycoside group in U8G-NEO hydrogen bonds with G9:pro-*S*p, similar as in N1-NEO. For the NEO and RIO complexes the contacts around A17 are similar regardless of the mutation.

### Comparison with NMR experiments

Solution NMR structures of the N1 riboswitch bound to RIO and PAR show similar interactions of the aminoglycoside 6′ group (20). Namely, in the NMR structure of the riboswitch with RIO, the 6′-N ammonium hydrogen bonds with the G9 phosphate and O4 of the U10 base (see Figure 5D and Supplemental Figure S17B). In the NMR structure with PAR, the corresponding 6′-OH hydroxyl holds the same contacts (Figure 5E). Our simulations confirmed the hydrogen bond between 6′-N and G9 phosphate in N1-RIO and N1-NEO but a corresponding bond between 6′-OH and G9 phosphate in N1-PAR was unstable even though it was present in the starting structure (Supplemental Table S7). The NMR data suggest that, regardless of the 6′-OH and G9 contact, the generally fewer contacts around the 6′-OH group influence the motions of A17 and destabilize the apical loop (20). Indeed, in our REMD simulations, the flexibility of A17 differs between the systems; the A17 base is strongly stabilized in the complexes with RIO and NEO, whereas in N1-PAR it maintains some mobility (Figure 2A).

The distances used as NMR restraints are coherent with most interatomic distances measured in REMD simulations (Supplemental Table S7). Overall, the RNA seems to be slightly more flexible than in NMR models, but this effect can be partly explained by the higher temperature applied in the simulations. Also, all major direct hydrogen bonds between aminoglycosides and RNA detected in the simulations correspond to restraints imposed in NMR, with one exception. The above mentioned hydrogen bond between 6′-OH and G9:pro-*S*p in the PAR complex is unstable in the simulations. The observed discrepancy between the NMR-derived structure and trajectories around the PAR 6′-OH group extends also to the contacts of A17:N6. In N1-PAR simulations, the A17 base may hydrogen bond not only with G5, but also, via its Hoogsteen edge, with the WC edge of U8 (Figure 3B). The possibility of this A17–U8 interaction, practically absent in the complexes with RIO and NEO (Figure 3 A), seems to allow the ribosome scanning of the N1-PAR mRNA complex because apparently makes A17 more flexible.

In the earlier 2010 NMR structure of the N1 riboswitch bound with RIO (PDB ID: 2kxm), the G9 phosphate is more than 6 Å from the 6′-N group, which is not in accord with the simulation-derived hydrogen bond network around the 6′ group (Supplemental Figure S17C). Contrarily, in the latest 2016 NMR structure (PDB ID: 2n0j), the same distance, between the 6′-N and G9:pro-*S*p, was confined to 3 Å (Supplemental Table S7). This observation reinforces the importance of the choice of NMR restraints during the refinement procedure and confirms that an MD simulation can assist in enhancing existing NMR models (49).

NMR imino proton solvent exchange experiments with the free riboswitch at 30°C (20) indicate destabilization of G5 (contrary to its stable positioning in the complexes with PAR and RIO). This is in agreement with the simulations; in the free riboswitch there is almost no pairing involving G5 whereas in the complexes frequent non-Watson–Crick pairing of G5 with A17 is observed (Figure 3). Thus, depriving A17 of the Hoogsteen edge hydrogen acceptors should lead to breaking of the G5–A17 contact and diminishing riboswitch activity. Indeed, the A17C riboswitch mutant shows reduced gene regulatory activity against NEO (50).

### Similarity between N1 riboswitch and A-site

**Figure 7.**
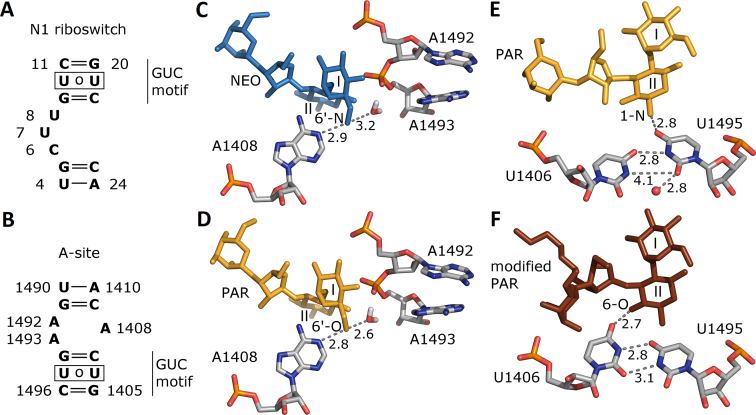
Sequences of the (**A**) N1 riboswitch and (**B**) bacterial decoding A-site. (**C**, **D**) Crucial contacts of aminoglycoside 6′ group within A-site from the crystal structures (NEO complex, PDB ID 2ET4 (51), PAR complex: 1J7T (52)) and optimization protocol with hydrogen atoms of (53). (**E**, **F**) Two conformations of U1406 and U1495 found in the crystal structures of the A-site – PAR complex, PDB ID 1J7T and A-site – modified PAR complex, PDB ID 2BEE (54). Distances between non-hydrogen atoms are in Å. One crystal water molecule found close to uridines in the PAR complex is shown as a red sphere.

Aminoglycosides bind to the bacterial rRNA decoding site in the small subunit (A-site). The sequences of the aminoglycoside binding A-site and N1 riboswitch are shown in Figure 7A and B. The neamine core of various aminoglycosides (ring I and II) is similarly positioned in both bulges. However, with respect to the neamine core, the riboswitch contains a GUC motif above the internal bulge and a GU pair below the bulge. Contrarily, in the A-site, the position of the neamine core is inverted with respect to the GUC motif. This bears out the finding that for aminoglycoside binding, apart from the electrostatic component (53), the shape and structural fit of the binding cleft are rather important (55).

Figure 7C and D shows the contacts of the NEO 6′-NH$_3^+$ and PAR 6′-OH groups in the crystal structures. Both groups are close to a water molecule and hydrogen bond with A1408 that is stably sandwiched between bases C1409 and G1494 via van der Waals interactions (56). Thus, in the A-site, the hydrogen bond between A1408 and the aminoglycoside is not essential for maintaining the A1408 stability. This is in accord with the fact that the A-site does not differentiate between NEO and PAR and both maintain extra-helical states of A1492 and A1493. In contrast, the 6′ group in the N1 riboswitch interacts with many RNA fragments: the G9 phosphate, the U10 carbonyl oxygen and nitrogen atoms of the labile A17 base. Thus, the change from a hydroxyl to an ammonium group at this position is expected to affect interactions with the riboswitch.

Shifting of Watson–Crick edge hydrogen bonds between the uridine pairs exists both in the riboswitch (U10$\circ$U21) and the A-site (U1406$\circ$U1495). In both systems, the uridines are in the vicinity of the aminoglycoside ring II. In the crystal structures of most of the aminoglycoside complexes with the A-site (including RIO, NEO, PAR), the conformation of the U1406 and U1495 pair is similar as in Figure 7E. In one structure, with modified PAR, the uracils are shifted (Figure 7F). In the A-site the uridine pair also switched conformations in MD simulations and was found to be crucial for stable aminoglycoside binding (57,58), similar to the situation in the N1 riboswitch as shown in Figure 4 and Supplemental Figure S11.

## CONCLUSION

**Figure 8.**
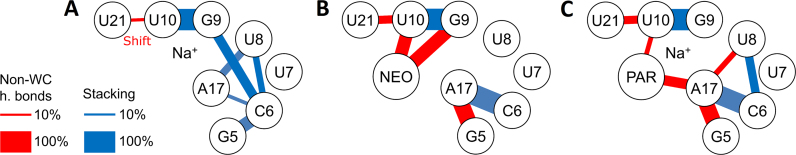
Schematic interaction map summarizing the major non-Watson–Crick hydrogen bonds and stacking interactions between selected bases and 6′ group of aminoglycosides in (**A**) the free riboswitch, (**B**) complex with NEO, (**C**) complex with PAR. The width of red and blue lines indicates the percentage of simulation frames in which these interactions were present. For clarity, the interactions observed in <10% of frames and the stable stacking between G5 and G9 in all simulations are omitted. High Na^+^ density and uridine shift are symbolically marked in relevant panels.

We performed all-atom MD and REMD simulations of the N1 riboswitch in the free form and in complexes with three 2-DOS aminoglycosides: NEO and RIO (both active *in vivo*), and PAR (inactive *in vivo*). A summary of our simulation results is schematically presented in Figure 8. We found that interactions within the RIO complex are very similar to those in the NEO system. We also observed that NEO and RIO stabilize neighboring riboswitch nucleotides via the 6′ ammonium group and similarly influence riboswitch dynamics. Contrarily, PAR allows for more flexibility in the bulge region because the contact with G9 is absent. Instead, a hydrogen bond between the A17 base and PAR 6′-OH is observed in the simulations. Even though this interaction is present for only ∼40% of simulation time, it occurred repeatedly and interchangeably together with a hydrogen bond between 6′-OH and U10. Interestingly, the N1-PAR complex partially mimics the conformations and interactions prevalent in the free riboswitch. Also, both systems have a dense pool of sodium ions at the same site.

The REMD simulations point to interactions that are not captured in NMR models, namely hydrogen bonds between A17 and U8, A17 and PAR, and stacking between U8 and C6 together with the flipped-in position of U8. The contacts used as NMR restraints in model refinement that are not preserved in the simulations include the hydrogen bond between PAR 6′-OH and G9:pro-*S*p. Introducing this contact in the simulation would lead to a completely different structural dynamics. Therefore, careful verification of the contacts in NMR structures is crucial for simulations, especially if the contact affects ligand binding.

The riboswitch shares several similar features with the A-site, such as the GUC motif and the uracil pair that shifts its WC edge hydrogen bonding pattern. However, the A-site does not recognize the subtle difference between NEO and PAR. We postulate that the reason why the riboswitch discriminates between these two aminoglycosides is the neighborhood of the 6′ group, especially the contact with the G9 phosphate, leading to different interactions and dynamics. In the A-site, the 6′ group stably contacts only the A1408 base in each complex but this base is also stable in the free A-site.

Overall, our work underlines the importance of atomistic-level details of the dynamics carried out on NMR models of nucleic acids. Such dynamics can be effectively extended with MD simulations using enhanced sampling in order to help design similar riboswitches and ligands for more accurate control of mRNA translation.

## Supplementary Material

Supplementary Data
