# Supplementary material for: Molecular mechanisms for dynamic regulation of N1 riboswitch by aminoglycosides
Source: Nucleic Acids Res. 2018 Sep 20;46(19):9960–70. doi: 10.1093/nar/gky833 (PMC6212780; doi:10.1093/nar/gky833)
Supplement: Supplementary Data [file gky833_supplemental_files.zip › SI-new20.pdf]

Molecular mechanisms for dynamic regulation of N1 riboswitch by aminoglycosides

Supporting Information

Marta Kulik [1,2], Takaharu Mori [1], Yuji Sugita [1,*] and Joanna Trylska [2,*]

[1]RIKEN, Hirosawa, Wako City, Saitama 351-0198, Japan
[2]Centre of New Technologies, University of Warsaw, Banacha 2c, 02-097 Warsaw, Poland

* To whom correspondence should be addressed. Yuji Sugita, Tel: +81 48 4621407; Fax: +81 48 4674532; Email: sugita@riken.jp and Joanna Trylska, Tel: +48 22 5543600; Fax: +48 22 5540801; Email: joanna@cent.uw.edu.pl

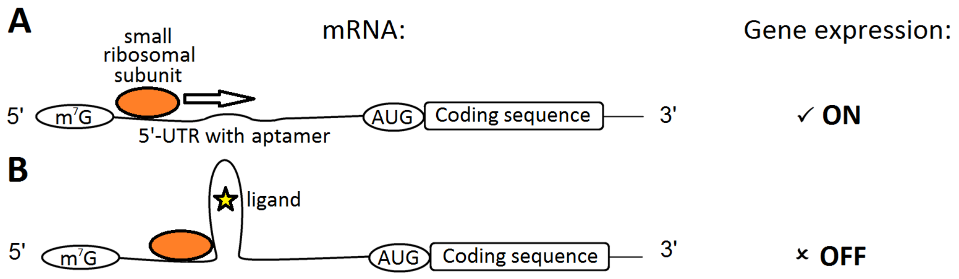

Figure S1: A scheme of the roadblock mechanism in the eukaryotic mRNA containing a translation regulating riboswitch. (**A**) The small ribosomal subunit proceeds from the 5'-terminus of mRNA towards the AUG start codon where it assembles with the large subunit to initiate translation. (**B**) Upon ligand binding to the aptamer, the small subunit is blocked before reaching the start codon and the mRNA coding sequence cannot be translated.

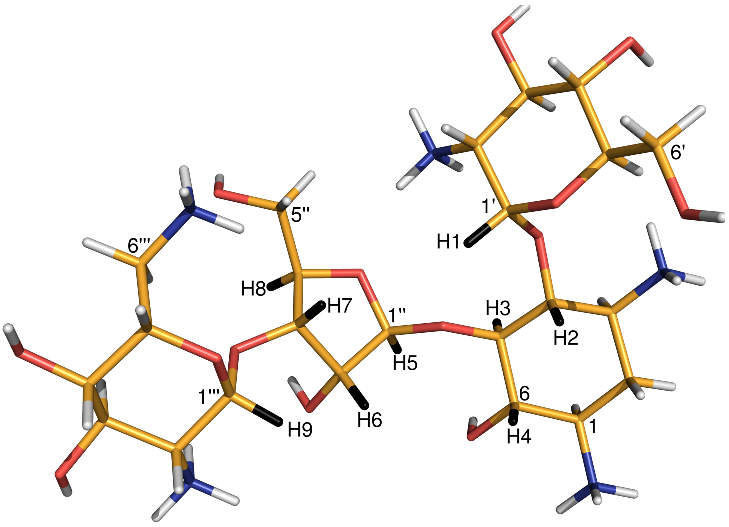

Figure S2: The starting structure of paromomycin in a conformation taken from the Protein Data Bank with ID 2mxs. The distances between protons that are marked black were compared with the distances from solution NMR experiments. The standard numbering of carbon atoms is shown.

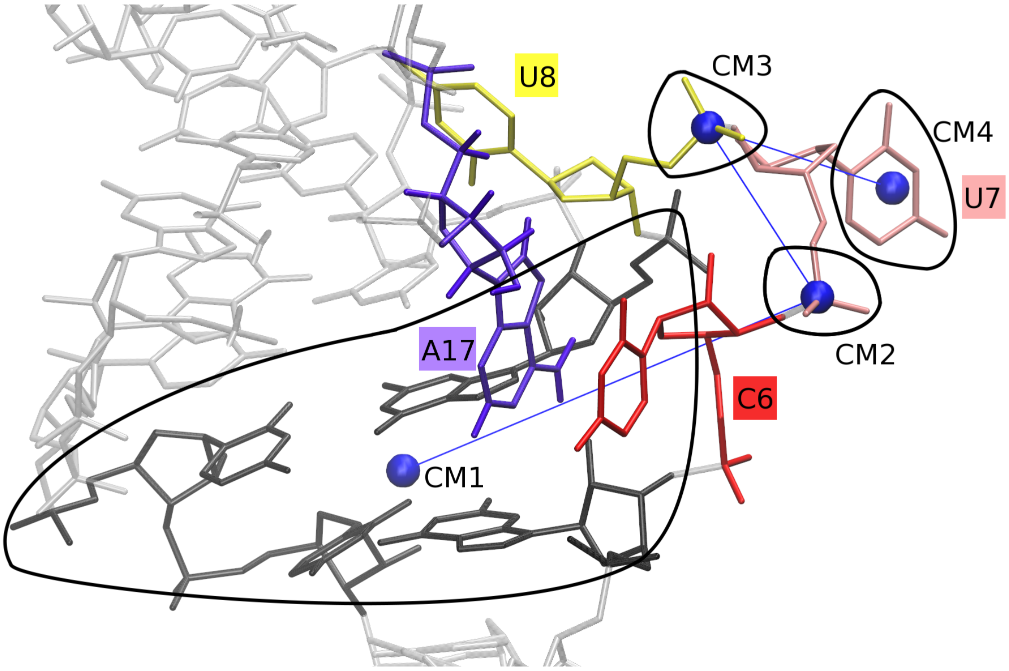

Figure S3: Definition of the pseudo-dihedral angle calculated to assess the flipping of bases. The example is given for the U7 base. CM1 represents the center of mass of nucleotides 5, 9, 22 and 23 marked in dark grey. CM2 and CM3 are the centers of masses of two phosphate groups neighboring U7 and CM4 is the center of mass of the U7 base. The definition was introduced by Song et al. [5]. For nucleotide numbering see Figure 1 of the main text.

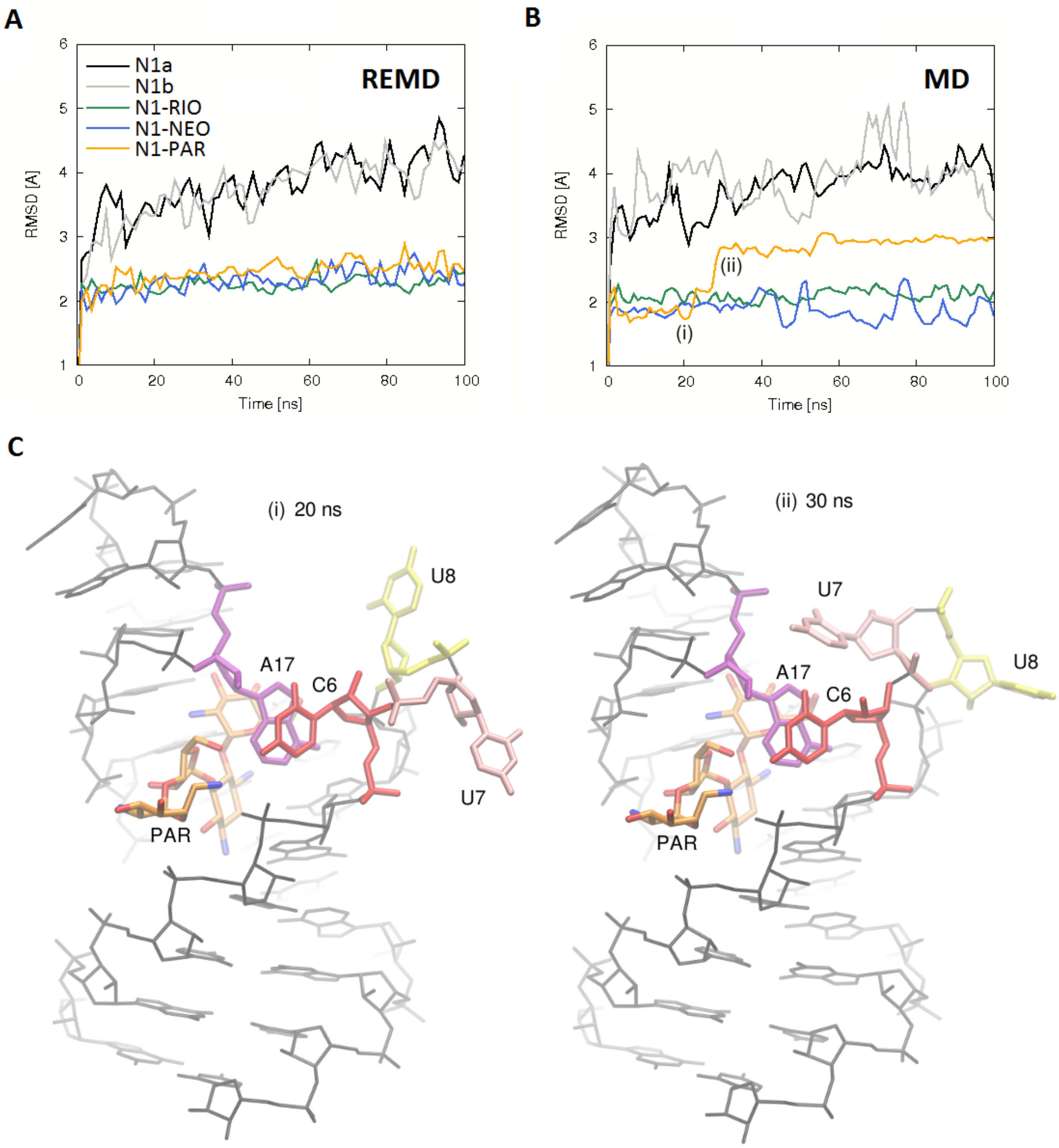

Figure S4: Root mean square deviation (RMSD) of RNA in the free N1 riboswitch and in the complexes with aminoglycosides in (**A**) REMD and (**B**) MD simulations. Data in the graphs were smoothed using the Bezier method. (**C**) Snapshots from MD simulations of the N1-PAR complex presenting the conformational change of the bulge bases between 20 ns and 30 ns. RMSD was calculated for RNA heavy atoms, after their least square fitting to the reference starting structure.

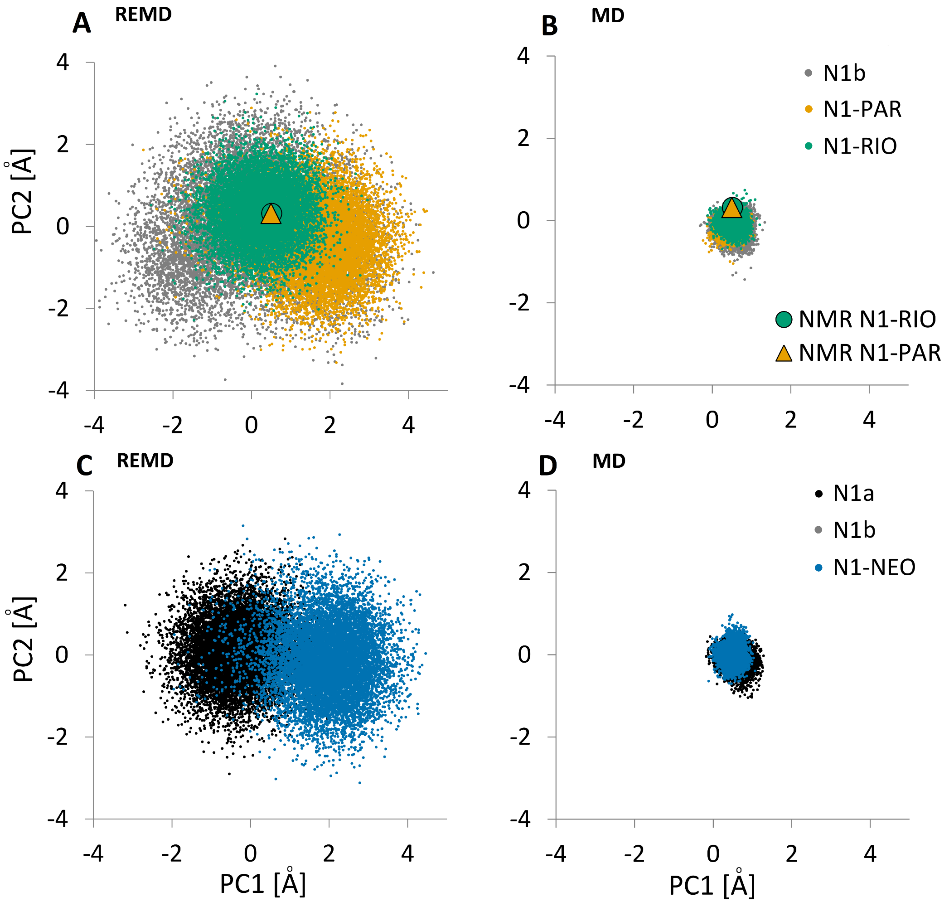

Figure S5: Principal components (PC) obtained in PCA using the dihedral angles of the RNA backbone (α, β, γ, δ, ϵ, ζ) and the glycosidic angle (χ). Due to major overlaps, the results are shown in four panels: (**A,C**) REMD at 311 K, (**B,D**) MD at 310 K. Two PCs with the lowest frequencies (PC1 and PC2) were projected on the eigenvectors derived for the N1b system at 311 K. Additionally, the positions of the starting NMR structures (2n0j for the N1-RIO system and 2mxs for the N1-PAR system) are marked with symbols. Naming of the simulations is in Table 1 of the main text.

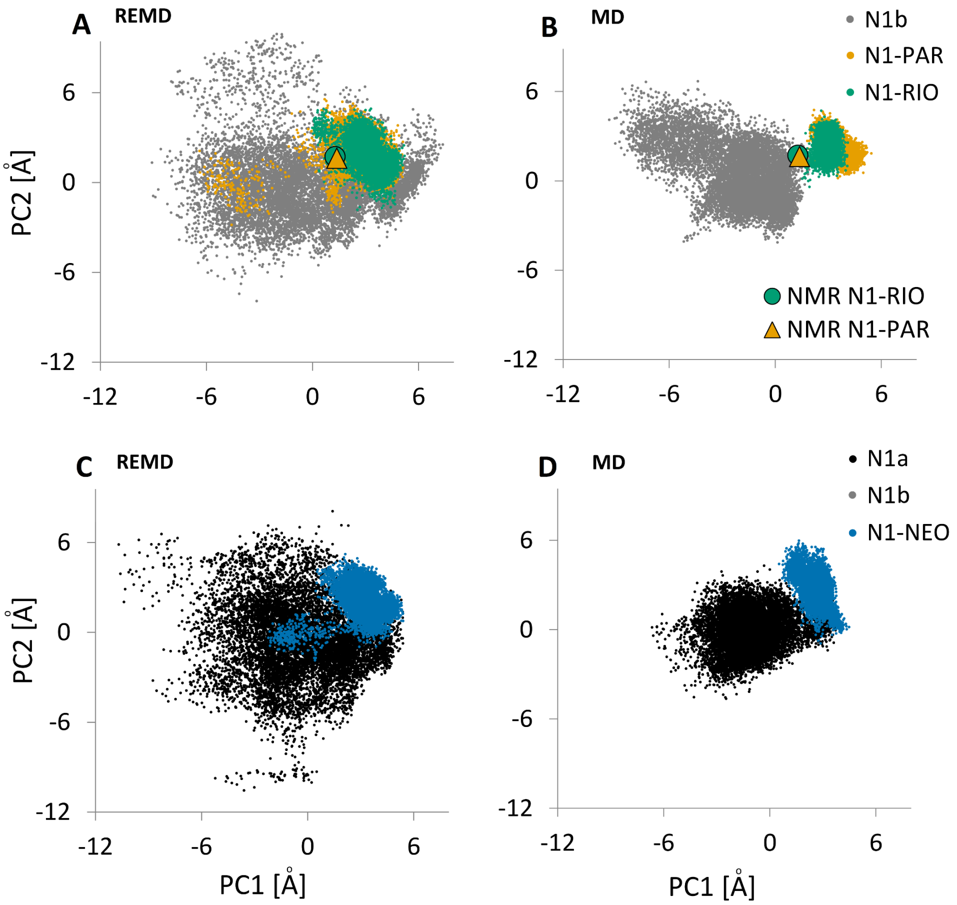

Figure S6: Principal components obtained using Cartesian coordinates of RNA from (**A,C**) REMD at 311 K, (**B,D**) MD at 310 K.Two PCs with the lowest frequencies (PC1 and PC2) were projected on the eigenvectors derived for the N1b system at 311 K. Additionally, the positions of the starting NMR structures (2n0j for the N1-RIO system and 2mxs for the N1-PAR system) are marked with symbols. Naming of the simulations is in Table 1 of the main text.

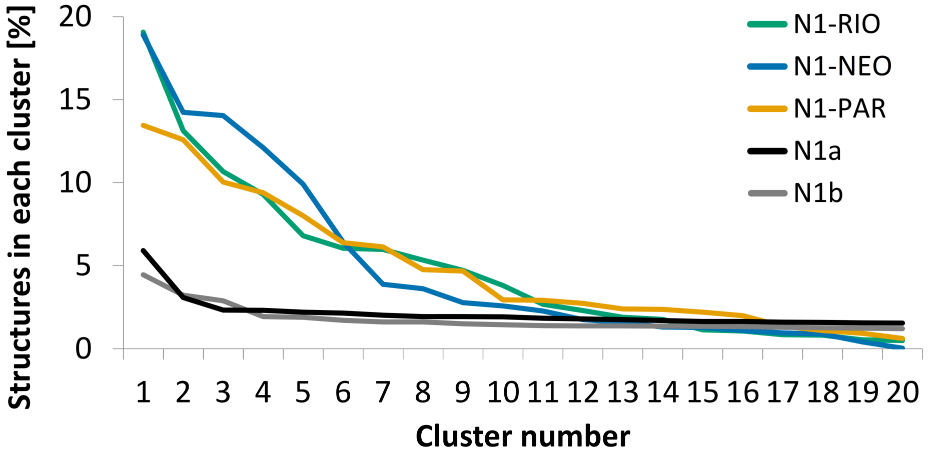

Figure S7: The percentage of conformations present in 20 most populated clusters.

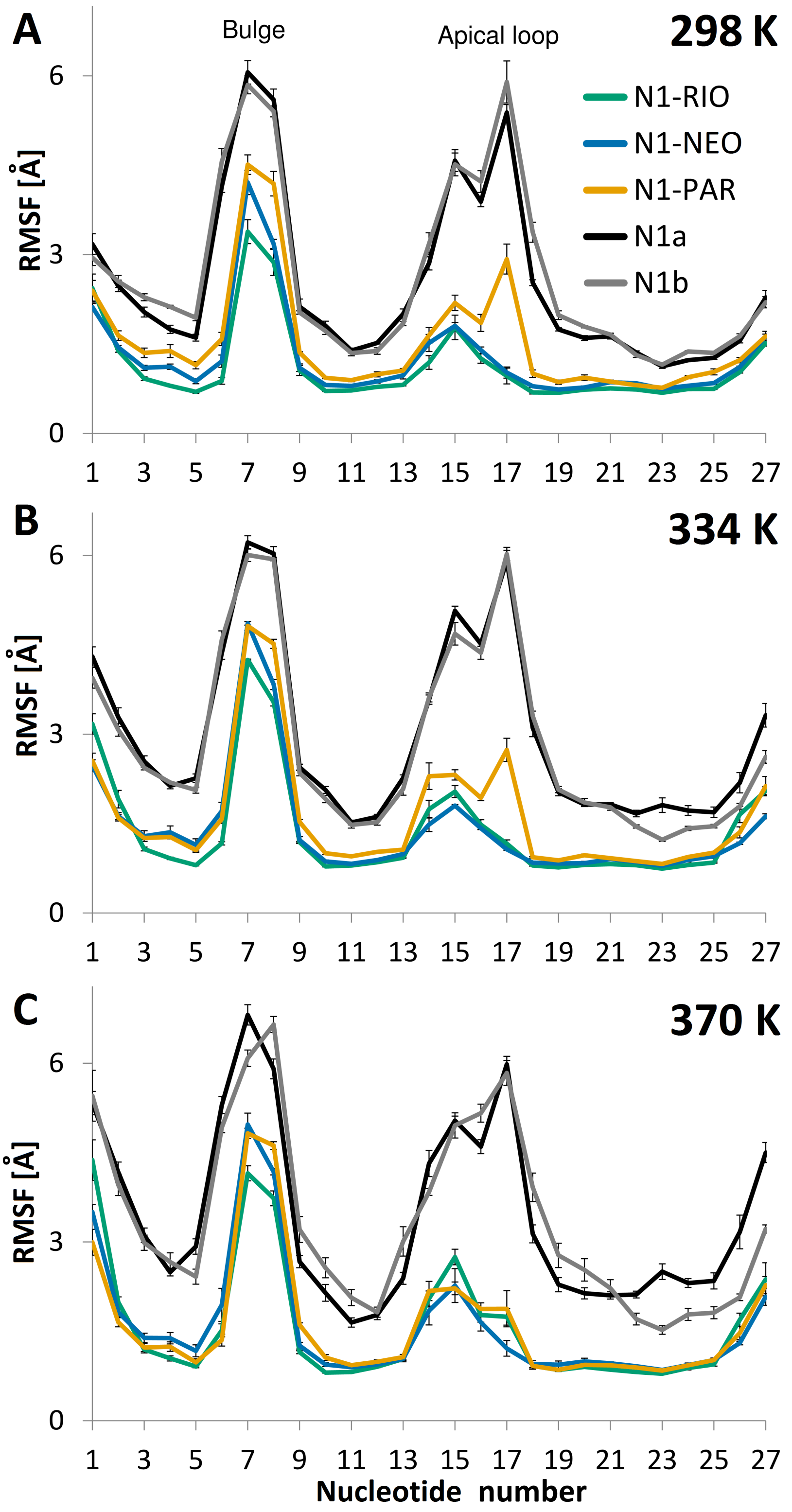

Figure S8: RMSF of RNA nucleotides in free riboswitches N1a and N1b and their complexes with aminoglycosides from REMD at 298 K, 334 K and 370 K (exact values: 298.15, 333.84 and 370.01 K, respectively). Errors were estimated by block averaging using 10 ns trajectory blocks.

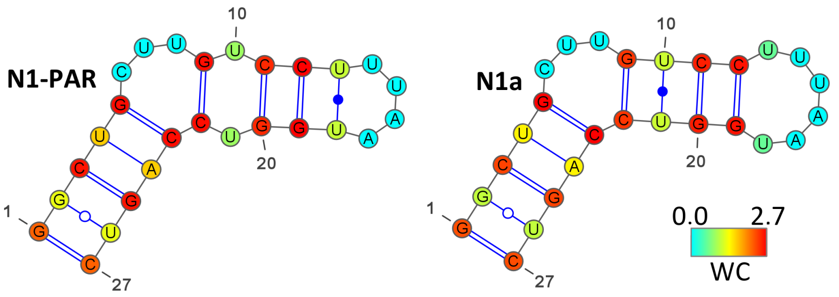

Figure S9: RNA secondary structure of the N1-PAR complex and free N1a riboswitch color-coded according to the average number of WC-edge base pairs. Schematic single and double bonds between A-U and G=C mark canonical WC bonds. Circles in between bases refer to hydrogen bonds that also use WC edges, but are non-canonical, according to Leontis and Westhof nomenclature [4].

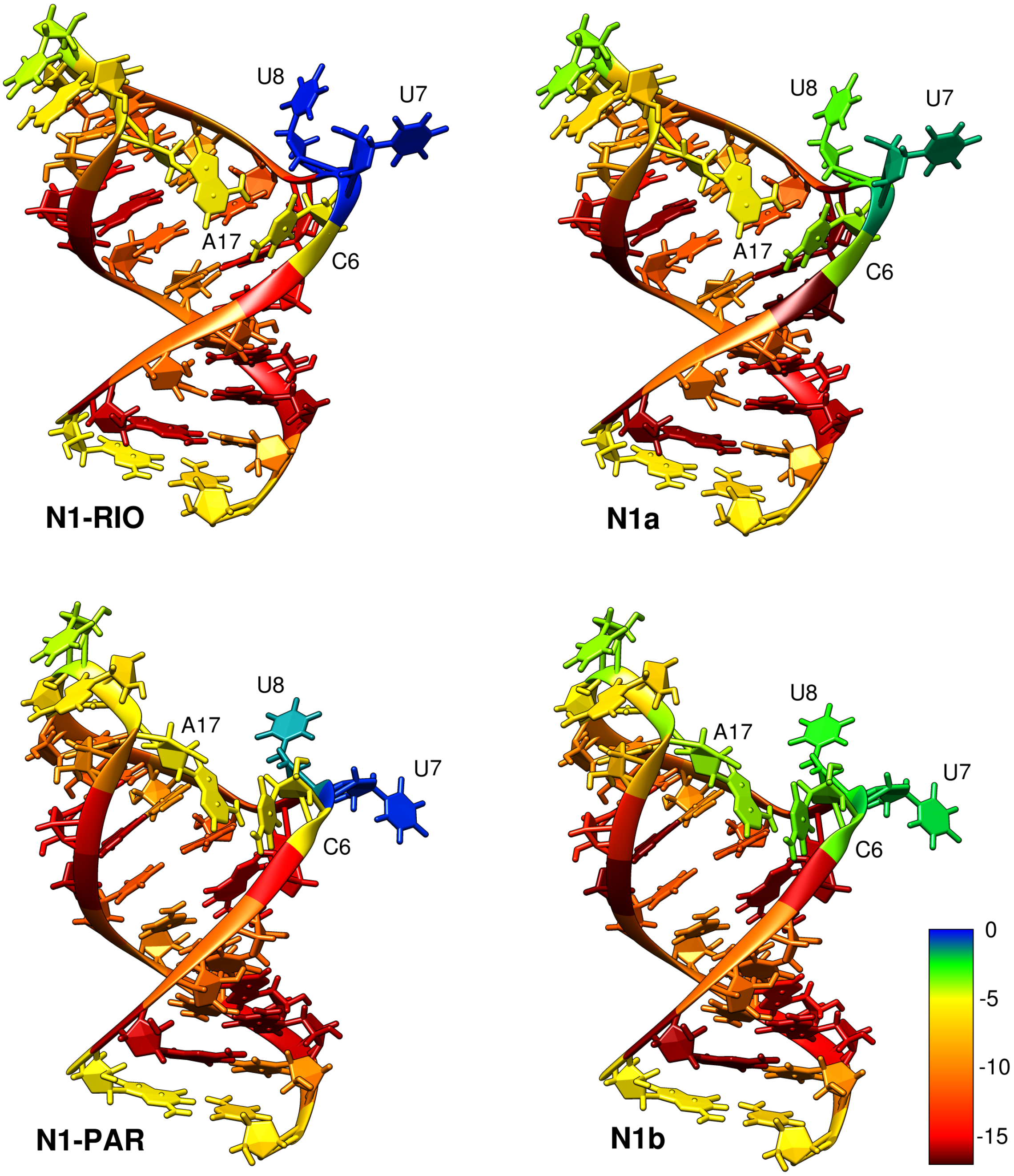

Figure S10: The N1 riboswitch color-coded according to the van der Waals contribution to stacking energy per base averaged over REMD trajectory. For clarity of the comparison, the energies are mapped on the starting structures (the first NMR structure 2n0j of N1 riboswitch in the complex with RIO and 2mxs with PAR). The scale is in kcal/mol.

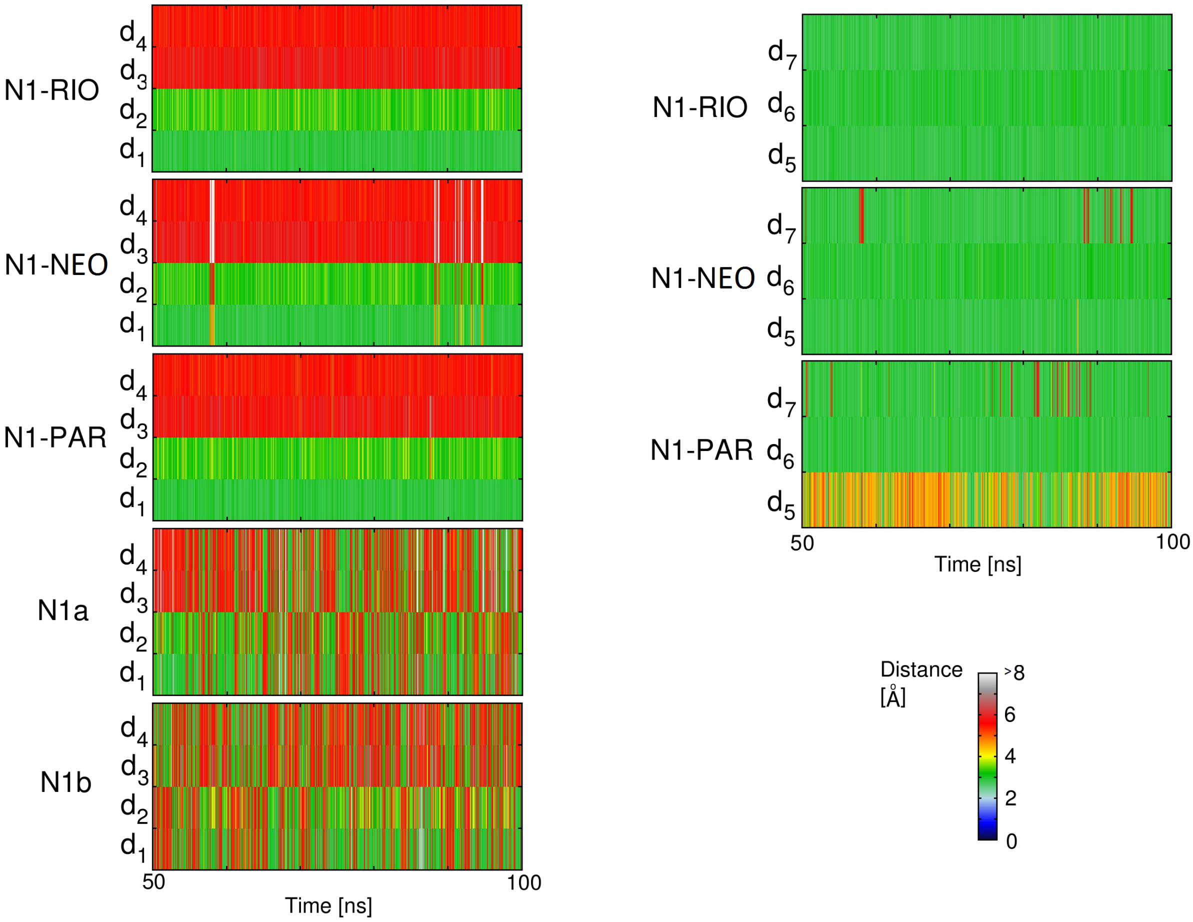

Figure S11: Distances between chosen uridine atoms and 6'-N/3-N atoms in the RIO/NEO complexes or 6'-O/3-N atoms in the PAR complex in time. For the definitions of the distances d_1–d_7 see Figure 4 in the main text.

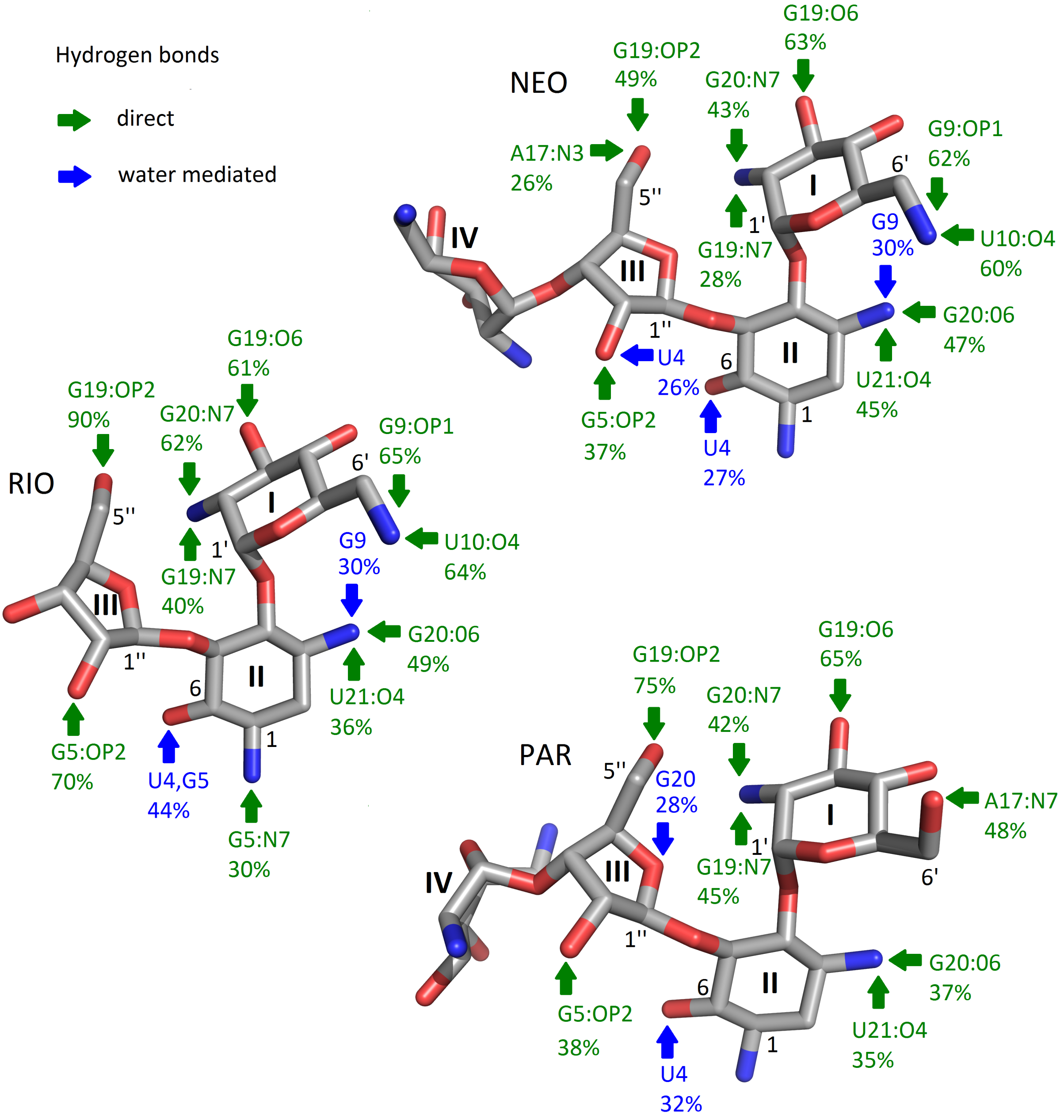

Figure S12: Direct (in green) and water mediated (in blue) aminoglycoside–RNA hydrogen bonds with the percentage of frames during which these bonds were formed in REMD simulation at 311 K. For ammonium groups serving as proton donors, the hydrogen bonds from all proton donors are summed. Only the interactions present in more than 25% of simulation time are shown. The conformations of aminoglycosides are taken from the most populated cluster representatives. Numbering of carbon atoms and rings is shown in black. The atom names OP1 and OP2 refer to pro-*S*p and pro-*R*p, respectively.

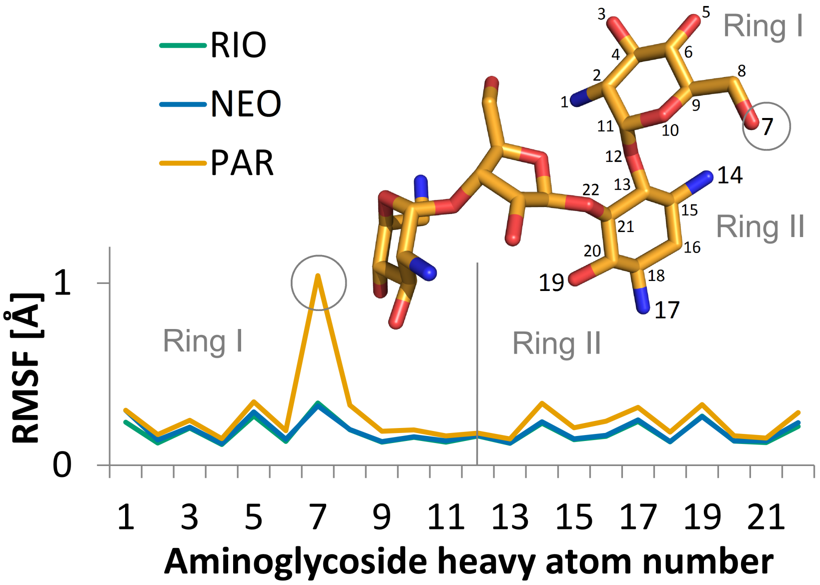

Figure S13: RMSF of aminoglycoside heavy atoms of the neamine core. The terminal 6'-OH group of paromomycin is circled.

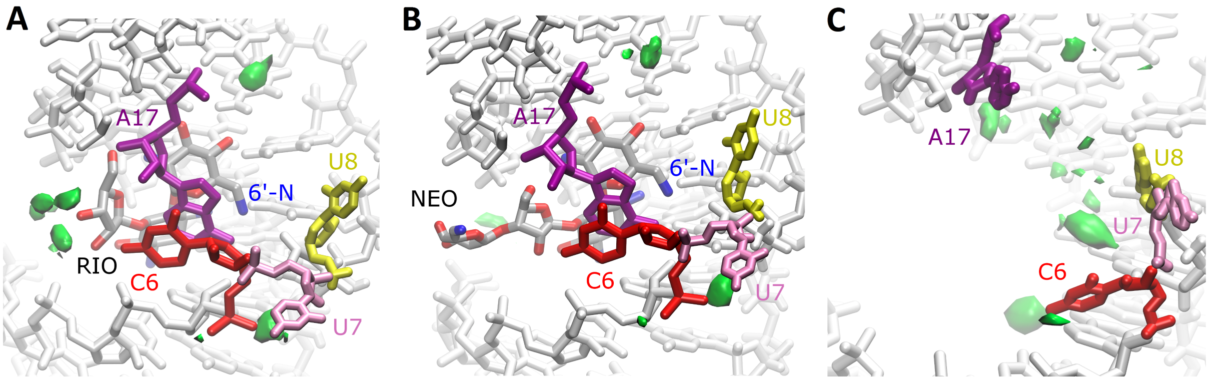

Figure S14: Ion density in the riboswitch complexes with (**A**) RIO, (**B**) NEO and (**C**) the free riboswitch – N1a. Areas of high Na^+ concentration (≥ 0.02 ions per $Å^3$) are shown as green surfaces. The structures of RNA and aminoglycosides are averaged over the trajectories.

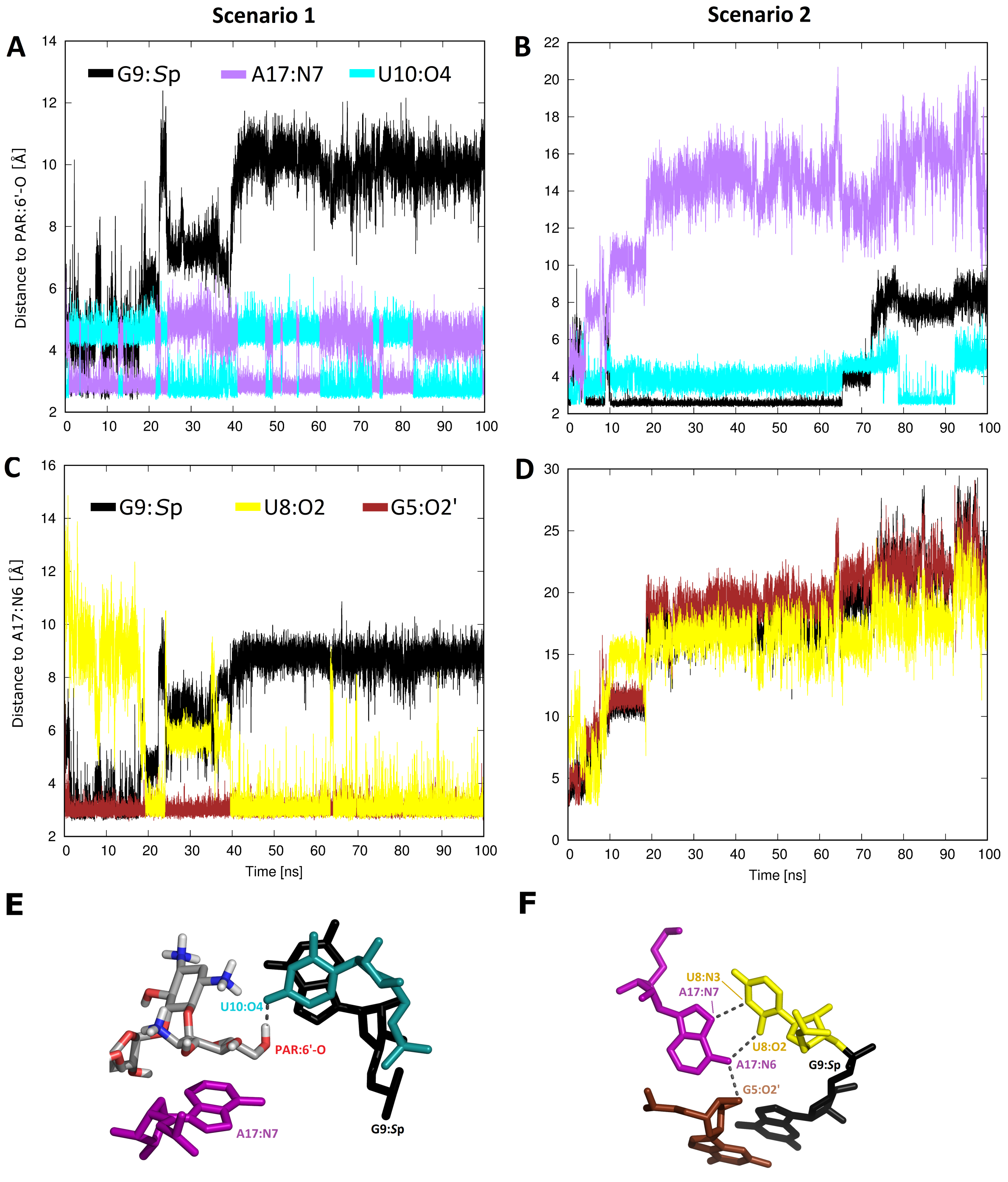

Figure S15: (**A,B**) Distances between the 6'-O atom in the N1-PAR complex with the atoms shown in the legend (the G9 phosphate oxygen, N7 atom of the A17 base, and O4 atom of the U10 base). (**C,D**) Distances between the N6 atom of the A17 base with the atoms shown in the legend (the G9 phosphate oxygen, the O2 atom of the U8 base and the sugar oxygen O2' of the G5 base). Panels (**A,C**) and (**B,D**) refer to the distances in time in two chosen REMD trajectories of N1-PAR, named Scenario 1 and 2, respectively. (**E,F**) The structural view and names of the atoms used for the calculation of the distances.

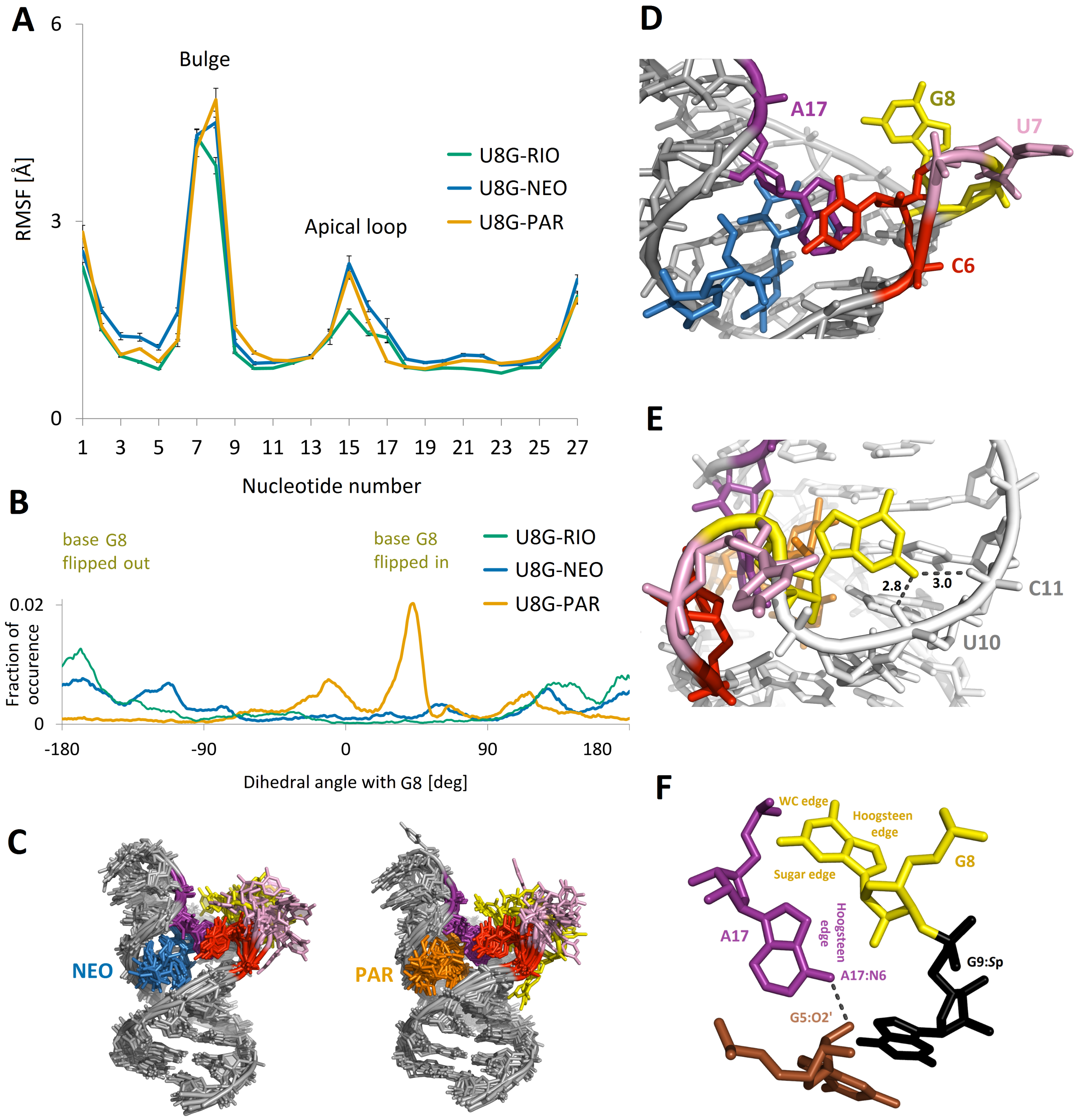

Figure S16: The results of the REMD simulations with the U8G mutation. (**A**) RMSF calculated per nucleotide. (**B**) Flipping of the G8 base. (**C**) Superposition of ten most populated clusters from the simulations of NEO and PAR complexes. (**D**) The orientation of the G8 base in the most populated cluster in the NEO complex. (**E**) Hydrogen bonds between the base G8 and phosphate oxygens in the most populated cluster in the PAR complex. (**F**) The structural view of A17 and G8 bases, showing the edges, in the second most populated cluster of the U8G-PAR simulations (compare with Figure S15F).

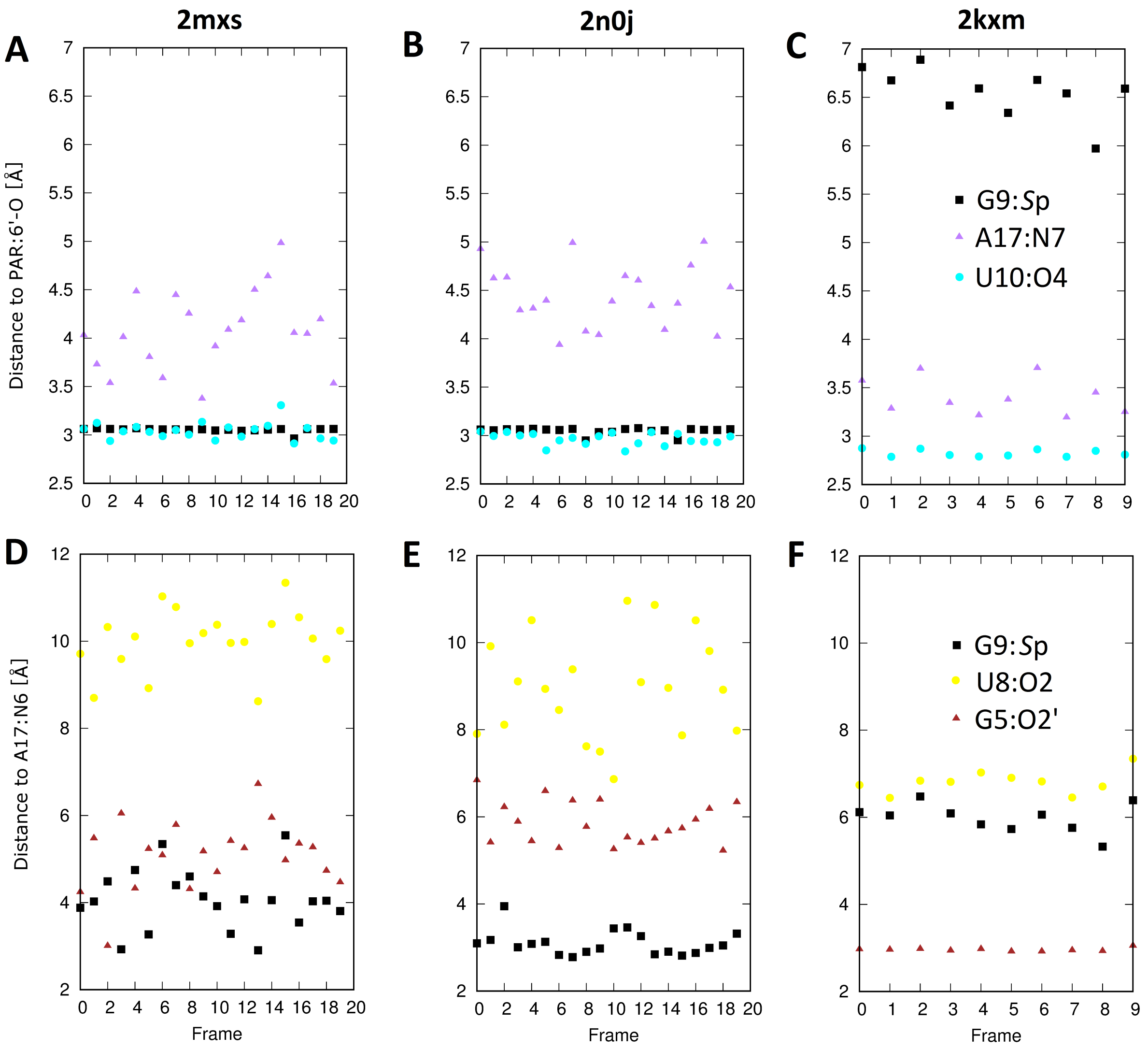

Figure S17: Distances in the NMR models. (**A,B,C**) Distances between the 6'-O atom in the N1-PAR complex or 6'-N in the N1-RIO complex with selected atoms marked in the legend (G9 phosphate oxygen, the N7 atom of A17 base and the O4 atom of the U10 base). (**D,E,F**) Distances between the N6 atom of the A17 base with selected atoms shown in the legend (G9 phosphate oxygen, the O2 atom of the U8 base and sugar oxygen O2' of G5). Panels (**A,D**), (**B,E**) and (**C,F**) refer to distances in different NMR models of the structures with PDB IDs 2mxs, 2n0j and 2kxm, respectively.

Table S1: Inter-proton distances obtained from NMR and MD simulations. The distances were measured in 100 ns MD simulations of free aminoglycosides (in explicit water and 100 mM NaCl) between the black labelled protons shown in Figure S2 and compared with the distances from the NMR studies [1] obtained for neomycin and ribostamycin. The NMR data for paromomycin are not available (n/a).

	NEO			PAR			RIO		
	NMR	MD		NMR	MD		NMR	MD	
	d [Å]	d [Å]	SD [Å]	d [Å]	d [Å]	SD [Å]	d [Å]	d [Å]	SD [Å]
H1 - H2	2.5	2.93	0.31	n/a	2.87	0.33	2.5	2.90	0.32
H1 - H3	3.0	3.01	0.36	n/a	3.12	0.46	3.1	3.00	0.42
H1 - H6	3.6	2.90	0.47	n/a	3.16	0.78	3.6	3.03	0.71
H1 - H8	>3.5	4.95	0.63	n/a	4.83	0.75	>3.5	4.92	0.70
H2 - H5	>4.0	4.49	0.18	n/a	4.42	0.48	>3.5	4.43	0.35
H3 - H5	2.3	3.03	0.50	n/a	2.82	0.46	2.2	3.01	0.48
H4 - H5	3.6	3.43	0.46	n/a	3.46	0.40	3.3	3.38	0.43
H4 - H6	3.3	4.70	0.36	n/a	4.63	0.42	3.1	4.60	0.41
H5 - H8	3.2	3.11	0.28	n/a	3.16	0.31	3.1	3.13	0.36
H6 - H9	3.1	4.38	0.41	n/a	4.25	0.52			
H7 - H9	2.6	2.71	0.28	n/a	2.70	0.31			
H8 - H9	3.9	4.05	0.29	n/a	4.07	0.34			

Table S2: The overlap between the covariant matrices calculated for the two halves of the analyzed parts of REMD simulations, namely between 50 – 75 ns and 75 – 100 ns. The calculation was performed using the Gromacs tools and the formulas for the overlap are as in gmx anaeig manual.

	N1-RIO	N1-NEO	N1-PAR	N1a	N1b
Normalized overlap	0.70	0.73	0.76	0.70	0.70
Shape overlap	0.70	0.74	0.76	0.70	0.70

Table S3: Watson-Crick hydrogen bond analysis showing the percentage of trajectory frames in which the indicated bases formed at least one pair via the Watson-Crick edge of each base (i.e. there was at least one hydrogen bond between two WC edges that could be uniquely assigned[a]). Only base pairs found in more than 2% of frames in at least one trajectory are presented, and, in addition, the pairs present in less than 50% of frames are in bold. All shown base pairs are in *cis* configuration. The first 50 ns of the simulation was omitted in this analysis.

		Percentage of simulation time [%]				
Base 1	Base 2	N1a	N1b	N1-RIO	N1-NEO	N1-PAR
G1	C27	89.1	87.9	86.4	89.2	85.1
G2	U26	50.9	50.8	64.2	65.2	64.5
C3	G25	85.4	86.7	96.6	96.5	95.4
U4	A24	80.8	63.3	92.6	92.2	92.5
G5	C23	97.3	97.7	98.5	97.9	98.2
G9	C22	89.0	93.9	98.6	98.7	98.5
U10	U21	57.9	55.8	**39.1**	**41.6**	**42.1**
C11	G20	88.7	89.9	97.9	98.0	96.4
C12	G19	90.6	96.6	99.0	99.0	99.0
U13	U18	**22.8**	**19.1**	57.6	57.8	53.8

[a] if there was only one hydrogen bond and some other edge was involved, e.g., the hydrogen bond was formed between the WC edge of one base and involved a corner atom of the second base that could be assigned to either WC or Hoogsteen edge (so it was impossible to assign the edge uniquely), such a pair was not classified as a WC/WC pair.

Table S4: Analysis of stacking between the RNA bases based on the van der Waals interactions. Following [3] we assume that two nucleobases are stacked if their van der Waals energy is lower than -0.5 kcal/mol. We show the percentage of trajectory frames in which the above criterion is met in at least 25% of frames and in at least one trajectory, except for the following pairs of bases with very stable (> 90% of frames) stacking in all systems: G1-G2; G2-C3; G2-C27; C3-U26; C3-U4; U4-G25; U4-G5; G5-G9; G5-A24; G9-U10; G9-C23; U10-C11; U10-C22; C11-C12; C11-U21; C12-U13; C12-G20; U13-U14; U13-G19; G19-G20; G20-U21; U21-C22; C22-C23; C23-A24; A24-G25; G25-U26; U26-C27. Data for the bulge bases and A17 are in bold font. The first 50 ns of the simulation was omitted in this analysis. For the riboswitch secondary structure see Figure 1 of the main text.

		Percentage of simulation time [%]				
Base 1	Base 2	N1a	N1b	N1-RIO	N1-NEO	N1-PAR
U4	C6	**25**	**17**	0	0	0
G5	G25	38	43	34	41	35
G5	C6	**61**	**35**	0	0	0
C6	U8	**29**	**38**	4	8	**39**
C6	G9	**44**	**23**	0	0	0
C6	A17	17	15	**97**	**95**	**89**
U8	A17	**35**	**14**	8	6	6
G9	A24	36	30	18	22	20
U14	U15	19	31	1	2	3
U14	A16	38	36	47	50	45
U14	U18	54	51	92	94	91
U15	A16	84	79	95	96	96
A16	A17	23	25	0	0	4
A16	U18	78	75	100	100	96
A17	U18	**31**	**39**	0	0	0
U18	G19	96	82	100	100	100

Table S5: Non-Watson-Crick hydrogen bond analysis performed in MINT [3] with a list of base pair types classified based on the nucleotide edges that participate in hydrogen bonding and *cis* or *trans* configuration. The percentage of trajectory frames during which the indicated type of base pair appeared is shown for different simulations. Motifs containing the A17 base are in bold font. Only pairs found in more than 25% of frames and in at least one trajectory are presented. The WC, Hoogsteen and Sugar type of base pairing indicate that the hydrogen bonds are created with the Watson-Crick edge, Hoogsteen edge and sugar edge of the nucleotide, respectively. The slash (/) separates bases and comma is used if more than one base edge is engaged in hydrogen bonding or the edge cannot be assigned uniquely. The first 50 ns of the simulation was omitted in this analysis.

				Percentage of simulation time [%]				
Base 1	Base 2	Pair type	Config.	N1a	N1b	N1-RIO	N1-NEO	N1-PAR
G5	A17	Sugar/WC,Hoogsteen	Trans	0.4	0.1	**77.3**	**73.5**	**72.3**
U8	A17	WC/Hoogsteen	Trans	0.3	0.0	1.6	4.1	**26.3**
U10	U21	WC/WC,Hoogsteen	Cis	15.0	19.6	42.2	38.4	37.8

Table S6: Non-Watson-Crick hydrogen bond analysis performed in MINT [3] for the simulations with the U8G mutation for the same base pairs as specified in Table S6. The percentage of trajectory frames during which the indicated type of base pair appeared is shown for different simulations. The WC, Hoogsteen and Sugar type of base pairing indicate that the hydrogen bonds are created with the Watson-Crick edge, Hoogsteen edge and sugar edge of the nucleotide, respectively. Slash (/) separates bases and comma is used if more than one base edge is engaged in hydrogen bonding or the edge cannot be assigned uniquely. First 50 ns of simulation was omitted in this analysis.

				Percentage of simulation time [%]		
Base 1	Base 2	Pair type	Config.	U8G-RIO	U8G-NEO	U8G-PAR
G5	A17	Sugar/WC,Hoogsteen	Trans	74.2	64.9	70.0
G8	A17	WC/Hoogsteen	Trans	3.0	0.0	0.0
U10	U21	WC/WC,Hoogsteen	Cis	41.1	41.1	33.6

Table S7: Restraints used in the NMR refinement in the work of Duchardt-Ferner et al. [2] along with the average distances in various NMR structures (PDB codes: 2mxs - N1 complex with paromomycin, 2n0j, 2kxm - N1 complexes with ribostamycin) and in the last 50 ns of REMD trajectories. Aminoglycoside name in the second column "Atom 2" refers to the complex for which the given "Limit" was imposed in the NMR refinement procedure. Standard deviations for averages are given in parentheses. All values are given in Å. The atom names OP1 and OP2 refer to pro-*S*p and pro-*R*p, respectively.

Restraints used in the NMR refinement			Average distances in NMR			Average distances in REMD		
Atom 1	Atom 2	Limit	2mxs	2n0j	2kxm	N1-PAR	N1-NEO	N1-RIO
G9:**O**P1	6'-**O/N** (PAR/RIO)	3.0	3.1 (0.0)	3.0 (0.0)	6.6 (0.2)	8.5 (2.1)	3.6 (1.7)	3.6 (1.9)
U10:**O**4	6'-**N** (RIO)	3.0	3.0 (0.1)	3.0 (0.1)	2.8 (0.0)	4.1 (0.9)	2.8 (0.2)	2.8 (0.1)
G19:**O**P2	5"-O**H** (PAR/RIO)	2.0	2.1 (0.0)	2.1 (0.0)	4.2 (0.6)	2.3 (1.4)	3.6 (2.1)	2.0 (1.4)
G19:**O**P2	5"-**O**H (PAR/RIO)	3.0	2.9 (0.1)	3.0 (0.0)	3.8 (0.2)	3.1 (1.0)	4.0 (1.6)	2.9 (1.0)
G19:**O**6	3'-O**H** (PAR/RIO)	2.0	2.1 (0.0)	2.1 (0.0)	2.7 (0.2)	1.9 (0.4)	1.9 (0.3)	1.9 (0.2)
G19:**O**6	3'-**O**H (PAR/RIO)	3.0	2.9 (0.0)	2.9 (0.0)	3.1 (0.1)	2.8 (0.3)	2.7 (0.2)	2.7 (0.1)
G20:**O**6	3-**N** (PAR)	3.0	3.0 (0.0)	2.9 (0.0)	3.1 (0.0)	3.1 (0.8)	2.9 (0.1)	2.9 (0.1)
G5:**O**6	1-**N** (RIO)	3.0	3.0 (0.0)	3.0 (0.0)	3.0 (0.0)	2.8 (0.7)	2.9 (0.4)	2.9 (0.2)
A16:O**H**2'	U18:**O**P1	2.2	1.8 (0.1)	1.7 (0.0)	2.0 (0.8)	3.2 (1.5)	3.0 (1.2)	2.9 (1.1)
A16:**O**H2'	U18:**O**P1	3.2	2.7 (0.1)	2.7 (0.0)	2.8 (0.3)	3.6 (1.3)	3.4 (0.9)	3.3 (0.8)
U14:N**H**3	A17:**O**P2	2.0	1.9 (0.1)	2.0 (0.1)	1.9 (0.0)	3.3 (3.2)	2.8 (2.3)	2.6 (2.5)
U14:**N**H3	A17:**O**P2	3.0	2.9 (0.1)	3.0 (0.1)	2.9 (0.0)	4.1 (2.8)	3.6 (2.0)	3.5 (2.3)
U14:O**H**2'	A16:**N**7	2.0	1.8 (0.0)	1.7 (0.0)	1.9 (0.0)	3.9 (1.7)	3.5 (1.3)	3.8 (1.6)
U14:**O**H2'	A16:**N**7	3.0	2.7 (0.0)	2.7 (0.0)	2.8 (0.0)	3.9 (1.4)	3.5 (0.8)	3.7 (1.3)

Movie S1. Visualisation of the trajectory of the N1 riboswitch in the complex with PAR of one of the replicas with temperature varying in time, called Scenario S1. A zoom view at the 6'-OH group of PAR (colored by atom types) is shown. The hydrogen bonds formed by the PAR 6'-OH group are indicated with the Sp of the G9 phosphate group (in black), with A17:N7 (in purple) and with U10:O4 (in cyan). The second part of the movie shows how the phosphate group of G9 shifts away from the aminoglycoside and U8 flips in (in yellow).

Movie S2. Visualisation of the trajectory of the N1 riboswitch in the complex with PAR of one of the replicas with temperature varying in time, called Scenario S2. The full view of the complex (RNA in light grey and PAR in atom colors) is shown. The G5 base is in brown, C6 in red, U7 in pink, U8 in yellow, and A17 in purple. In the movie, the A17 base breaks the bond with G5 and flips outside the helix.

References

[1] Juan Luis Asensio, Ana Hidalgo, Igor Cuesta, Carlos González, Javier Cañada, Cristina Vicent, Jose Luis Chiara, Gabriel Cuevas, and Jesús Jiménez-Barbero. Experimental evidence for the existence of non-exo-anomeric conformations in branched oligosaccharides: Nmr analysis of the structure and dynamics of aminoglycosides of the neomycin family. Chemistry – A European Journal, 8(22):5228–5240, 2002.

[2] E. Duchardt-Ferner, S. R. Gottstein-Schmidtke, J. E. Weigand, O. Ohlenschläger, J. P. Wurm, C. Hammann, B. Suess, and J. Wöhnert. What a Difference an OH Makes: Conformational Dynamics as the Basis for the Ligand Specificity of the Neomycin-Sensing Riboswitch. Angew. Chem. Int. Ed. Engl., 55(4):1527–1530, Jan 2016.

[3] A. Górska, M. Jasiński, and J. Trylska. MINT: software to identify motifs and short-range interactions in trajectories of nucleic acids. Nucleic Acids Res., 43(17):e114, Sep 2015.

[4] N. B. Leontis and E. Westhof. Geometric nomenclature and classification of RNA base pairs. RNA, 7(4):499–512, Apr 2001.

[5] K. Song, A. J. Campbell, C. Bergonzo, C. de Los Santos, A. P. Grollman, and C. Simmerling. An Improved Reaction Coordinate for Nucleic Acid Base Flipping Studies. J. Chem. Theory Comput., 5(11):3105–3113, Nov 2009.
